# Nicotinamide nucleotide transhydrogenase regulates mitochondrial metabolism in NSCLC through maintenance of Fe-S protein function

**DOI:** 10.1084/jem.20191689

**Published:** 2020-03-20

**Authors:** Nathan P. Ward, Yun Pyo Kang, Aimee Falzone, Theresa A. Boyle, Gina M. DeNicola

**Affiliations:** 1 Department of Cancer Physiology, Moffitt Cancer Center, Tampa, FL; 2 Department of Molecular Pathology, Moffitt Cancer Center, Tampa, FL

## Abstract

Human lung tumors exhibit robust and complex mitochondrial metabolism, likely precipitated by the highly oxygenated nature of pulmonary tissue. As ROS generation is a byproduct of this metabolism, reducing power in the form of nicotinamide adenine dinucleotide phosphate (NADPH) is required to mitigate oxidative stress in response to this heightened mitochondrial activity. Nicotinamide nucleotide transhydrogenase (NNT) is known to sustain mitochondrial antioxidant capacity through the generation of NADPH; however, its function in non-small cell lung cancer (NSCLC) has not been established. We found that NNT expression significantly enhances tumor formation and aggressiveness in mouse models of lung tumor initiation and progression. We further show that NNT loss elicits mitochondrial dysfunction independent of substantial increases in oxidative stress, but rather marked by the diminished activities of proteins dependent on resident iron-sulfur clusters. These defects were associated with both NADPH availability and ROS accumulation, suggesting that NNT serves a specific role in mitigating the oxidation of these critical protein cofactors.

## Teaser

Human lung tumors engage in substantial mitochondrial metabolism. Here, Ward et al. demonstrate that nicotinamide nucleotide transhydrogenase (NNT) supports oxidative metabolism in NSCLC by mitigating Fe-S cluster oxidation.

## Graphical Abstract

**Figure:**
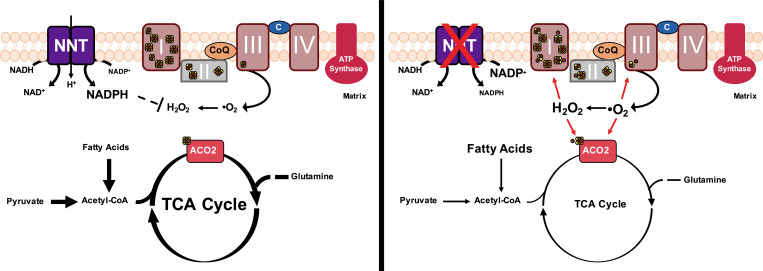

## Introduction

Metabolic rewiring facilitates the diversion of intermediate metabolites into pathways that supply the macromolecular determinants of the unbridled growth associated with human tumors. It is now appreciated that in addition to hallmark Warburg metabolism, many tumor species require substantial mitochondrial metabolism to thrive (Porporato et al., 2018). Indeed, non-small cell lung cancer (NSCLC) exhibits a simultaneous engagement of both glycolytic and oxidative metabolism (Fan et al., 2009). This increase in mitochondrial oxidation is supported by enhanced expression of pyruvate carboxylase, which provides auxiliary entry of glucose carbon into the TCA cycle in addition to that via pyruvate dehydrogenase (PDH; Sellers et al., 2015; Davidson et al., 2016). These activities support glucose oxidation in human lung tumors that exceeds that of normal adjacent lung (Hensley et al., 2016). Moreover, human lung tumors exhibit remarkable plasticity in oxidative fuel usage that is correlated with oxygen tension, indicating robust mitochondrial function (Hensley et al., 2016; Faubert et al., 2017).

Mitochondrial redox metabolism harnesses the potential energy stored in the reducing equivalents nicotinamide adenine dinucleotide (NADH) and flavin adenine dinucleotide which are generated from the catabolism of various carbon substrates (i.e., glucose, amino acids, fatty acids). These are then oxidized by multiprotein complexes of the electron transport chain (ETC), which couples the transfer of electrons to molecular oxygen with the generation of a proton gradient. This proton gradient is exploited by ATP synthase to generate useable energy for the cell in the form of ATP. Critical to ETC functionality is the maintenance of mitochondrial reducing power in the form of nicotinamide adenine dinucleotide phosphate (NADPH). NADPH is required for the detoxification of harmful ROS that are natural byproducts of oxidative metabolism (Navarro et al., 2017). ROS detoxification prevents the oxidation of proteins critical to metabolism, including the four respiratory chain protein complexes (I–IV) of the ETC, as well as other macromolecules such as the constituent lipid species of the inner mitochondrial membrane (IMM). Additionally, ROS detoxification protects against the oxidation of iron-sulfur (Fe-S) clusters, redox sensitive cofactors assembled within the mitochondria and incorporated into recipient Fe-S proteins that facilitate diverse functions such as DNA replication and repair, protein translation, and metabolism (Flint et al., 1993; Alhebshi et al., 2012; Rouault, 2015). Mitochondrial metabolism is particularly dependent on these clusters as fatty acid catabolism, respiration, and cofactor synthesis (e.g., lipoic acid, heme) all rely on Fe-S proteins (Lill and Mühlenhoff, 2008).

There are several sources of mitochondrial NADPH, including serine-dependent one-carbon metabolism (Ducker et al., 2016), isocitrate dehydrogenase (Jiang et al., 2016), malic enzyme (Ren et al., 2014), and the nicotinamide nucleotide transhydrogenase (NNT). NNT is an integral membrane protein associated with the IMM that harnesses the proton gradient across the membrane to couple the oxidation of NADH to the reduction of NADP^+^, yielding NADPH (Rydström, 2006; Kampjut and Sazanov, 2019). In bacteria, transhydrogenase activity accounts for up to 45% of total NADPH, which has led to the assertion that NNT should be an equal or greater contributor to the mitochondrial pool in mammalian species (Sauer et al., 2004; Rydström, 2006). Indeed, in the liver of mice, nearly 50% of the mitochondrial NADPH pool is sensitive to IMM uncoupling, indicative of a significant role for NNT (Klingenberg and Slenczka, 1959). Through subsequent studies, NNT has been established as a significant contributor to the mitochondrial NADPH pool (Fisher-Wellman et al., 2015; Ronchi et al., 2016; Francisco et al., 2018). The importance of NNT has also been evaluated in malignancy, where NNT is shown to contribute to the maintenance of redox homeostasis in several cancers (Gameiro et al., 2013; Ho et al., 2017; Chortis et al., 2018; Li et al., 2018).

While the maintenance of mitochondrial NADPH has been shown to be critical to lung tumor growth (Ren et al., 2014; Jiang et al., 2016), the contribution of NNT to lung tumorigenesis has not been evaluated. Herein, we provide the first evidence that NNT is a significant contributor to the mitochondrial NADPH pool in NSCLC and important for lung tumorigenesis. We show that loss of NNT activity disrupts mitochondrial metabolism in part through diminished Fe-S protein function. Our data indicate a more nuanced role for NNT in NSCLC redox homeostasis through the prevention of Fe-S cluster oxidation rather than protecting against global mitochondrial oxidation.

## Results

### Nnt supports lung tumorigenesis

Many common conditional lung tumor mouse models were generated from breeding strategies that employed C57BL6/J mice (Jackson et al., 2001, 2005; Meuwissen and Berns, 2005). Interestingly, these mice carry a homozygous in-frame deletion of exons 7–11 as well as a missense mutation in the mitochondrial leader sequence of the *Nnt* gene that result in the expression of a nonfunctional protein (Toye et al., 2005). Therefore, to assess the contribution of NNT to lung tumorigenesis, we exploited this natural knockout allele (*Nnt*^*Δex7-11*^).

**Figure 1.**
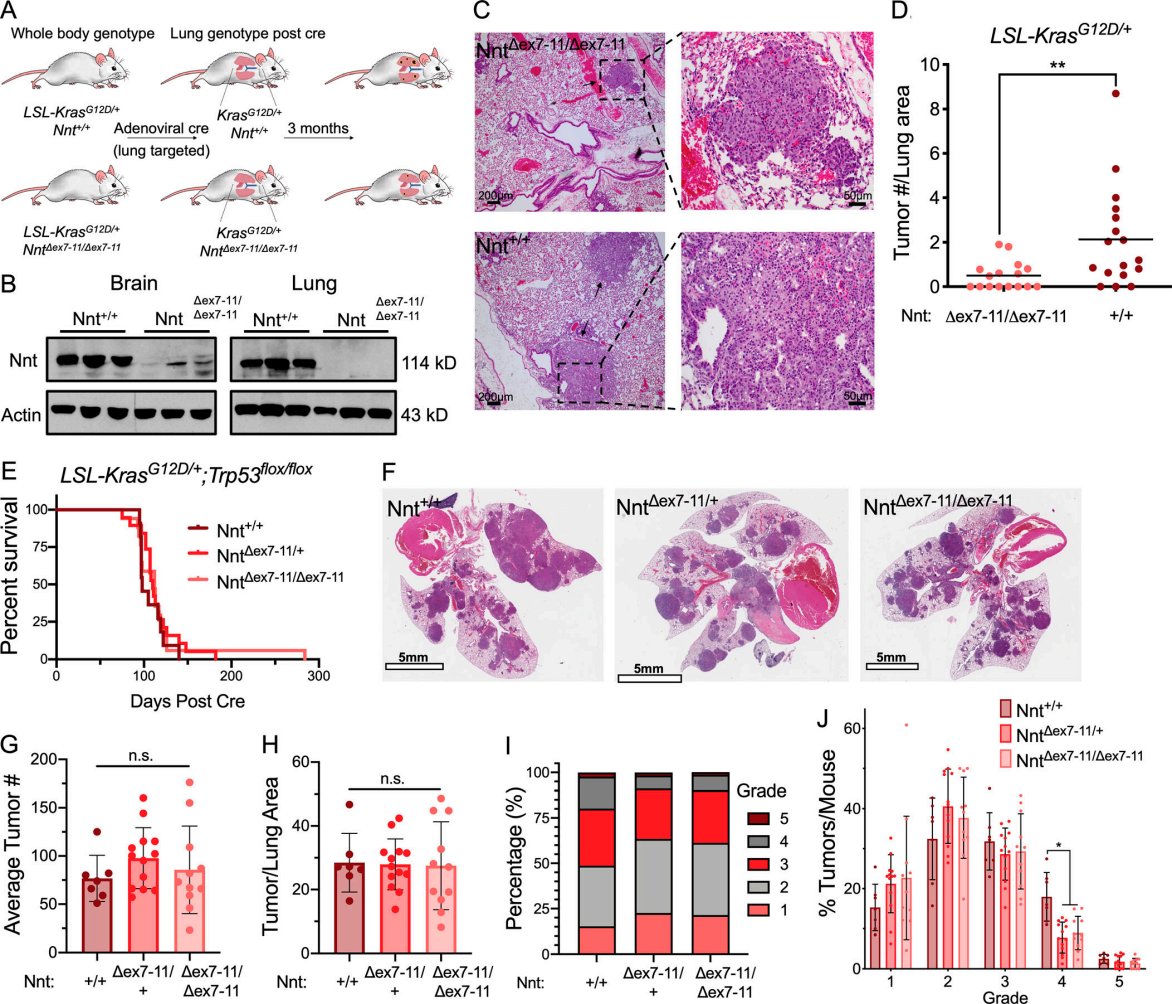
**NNT supports lung tumorigenesis. (A)** Schematic of *LSL-Kras^G12D/+^* mice genetics before and after adenoviral Cre administration. **(B)** Immunoblot analysis of Nnt and actin (loading control) expression in brain and lung lysates from *LSL-Kras^G12D/+^;Nnt^+/+^* and *LSL-Kras^G12D/+^; Nnt^Δex7-11/Δex7-11^* mice. **(C)** Representative H&E-stained lung sections of *LSL-Kras*^*G12D/+*^*; Nnt*^*Δex7-11/Δex7-11*^* and LSL-Kras*^*G12D/+*^*; Nnt*^*+/+*^ mice 3 mo after Cre induction. Left panel are 4×, bars represent 200 µm; right panel are 20×, bars represent 50 µm. **(D)** Average tumor number per lung area in *LSL-Kras*^*G12D/+*^*; Nnt*^*Δex7-11/Δex7-11*^ (*n* = 16) and *LSL-Kras*^*G12D/+*^*; Nnt*^*+/+*^ (*n* = 17) specimens collected at 3 mo (Student’s *t* test). **(E)** Survival rates of *LSL-Kras*^*G12D/+*^*; Trp53*^*flox/flox*^*; Nnt*^*+/+*^ (*n* = 11), *LSL-Kras*^*G12D/+*^*; Trp53*^*flox/flox*^*; Nnt*^*Δex7-11/+*^ (*n* = 19), and *LSL-Kras*^*G12D/+*^*; Trp53*^*flox/flox*^*; Nnt*^*Δex7-11/Δex7-11*^ (*n* = 17) mice following Cre induction (log-rank test). **(F)** Representative H&E-stained lung sections of *LSL-Kras*^*G12D/+*^*; Trp53*^*flox/flox*^*; Nnt*^*+/+*^ (left), *LSL-Kras*^*G12D/+*^*; Trp53*^*flox/flox*^*; Nnt*^*Δex7-11/+*^**(middle), and *LSL-Kras*^*G12D/+*^*; Trp53*^*flox/flox*^*; Nnt*^*Δex7-11/Δex7-11*^ (right) mice at experimental endpoint. Bars, 5 mm. **(G)** Average tumor number per each lung specimen (one-way ANOVA). **(H)** Fraction of total lung that was burdened by tumor (one-way ANOVA). **(I)** Distribution of tumor grades across all tumors for Kras^G12D/+^; p53^Δ/Δ^; Nnt^+/+^ (*n* = 530 tumors), Kras^G12D/+^; p53^Δ/Δ^; Nnt^Δex7-11/+^ (*n* = 1,269 tumors), and Kras^G12D/+^; p53^Δ/Δ^; Nnt^Δex7-11/Δex7-11^ (*n* = 943 tumors). **(J)** Average frequency of each tumor grade per mouse (two-way ANOVA). For G, H, and J, LSL-Kras^G12D/+^; Trp53^flox/flox^; Nnt^+/+^ (*n* = 7), LSL-Kras^G12D/+^; Trp53^flox/flox^; Nnt^Δex7-11/+^ (*n* = 13), and LSL-Kras^G12D/+^; Trp53^flox/flox^; Nnt^Δex7-11/Δex7-11^ (*n* = 11). For D, G, H, and J, data represented as mean ± SD. n.s., not significant; *, P < 0.05; **, P < 0.01.

First, we used a lung tumor model driven by mutant Kras (*LSL-Kras^G12D/+^*) to examine the influence of Nnt expression on lung tumor initiation. Infection of *LSL-Kras^G12D/+^* mice with adenovirus encoding Cre recombinase induces the expression of Kras^G12D^ in the lung epithelium and the formation of lung adenomas that rarely progress to higher-grade tumors (Jackson et al., 2001). We crossed these *LSL-Kras^G12D/+^* mice with C57BL6/J mice to generate cohorts with (*Nnt*^*+/+*^) or without Nnt expression (*Nnt*^*Δex7-11/Δex7-11*^) throughout the animal (Fig. 1, A and B). We found that expression of Nnt in this model resulted in significantly greater tumor burden 3 mo following Cre recombinase induction (Fig. 1, C and D). Next, we used a lung tumor progression model that is driven by concomitant expression of mutant Kras and p53 deletion (*LSL-Kras^G12D^*^/+^; *Trp53^flox/flox^*, also known as KP). Nnt expression did not alter the survival of KP mice following Cre-induction (Fig. 1 E). Interestingly, p53 deletion abrogated the effects of Nnt expression on lung tumor formation in this model, with substantial tumor burden present at the experimental endpoint regardless of Nnt status (Fig. 1 F). Quantification of the fraction of burdened lung demonstrated no difference across genotypes (Figs. 1, G and H).

While there were no differences in tumor burden between genotypes, we did observe differences in tumor aggressiveness as defined previously for this model (Jackson et al., 2005; DuPage et al., 2009). We found that 51.3% of tumors from Nnt^+/+^ mice were of grade 3 (adenocarcinoma) or greater, whereas only 36.5% and 38.8% of tumors from Nnt^Δex7-11/+^ and Nnt^Δex7-11/Δex7-11^ mice were high-grade (Fig. 1 I). This shift in tumor aggressiveness was evidenced by a significant increase in the frequency of grade 4 tumors in Nnt^+/+^ mice (Fig. 1 J). Collectively, these data indicate that Nnt promotes both lung tumor initiation and tumor aggressiveness.

### NNT loss does not compromise the mitochondrial thioredoxin (TXN) antioxidant system

**Figure S1.**
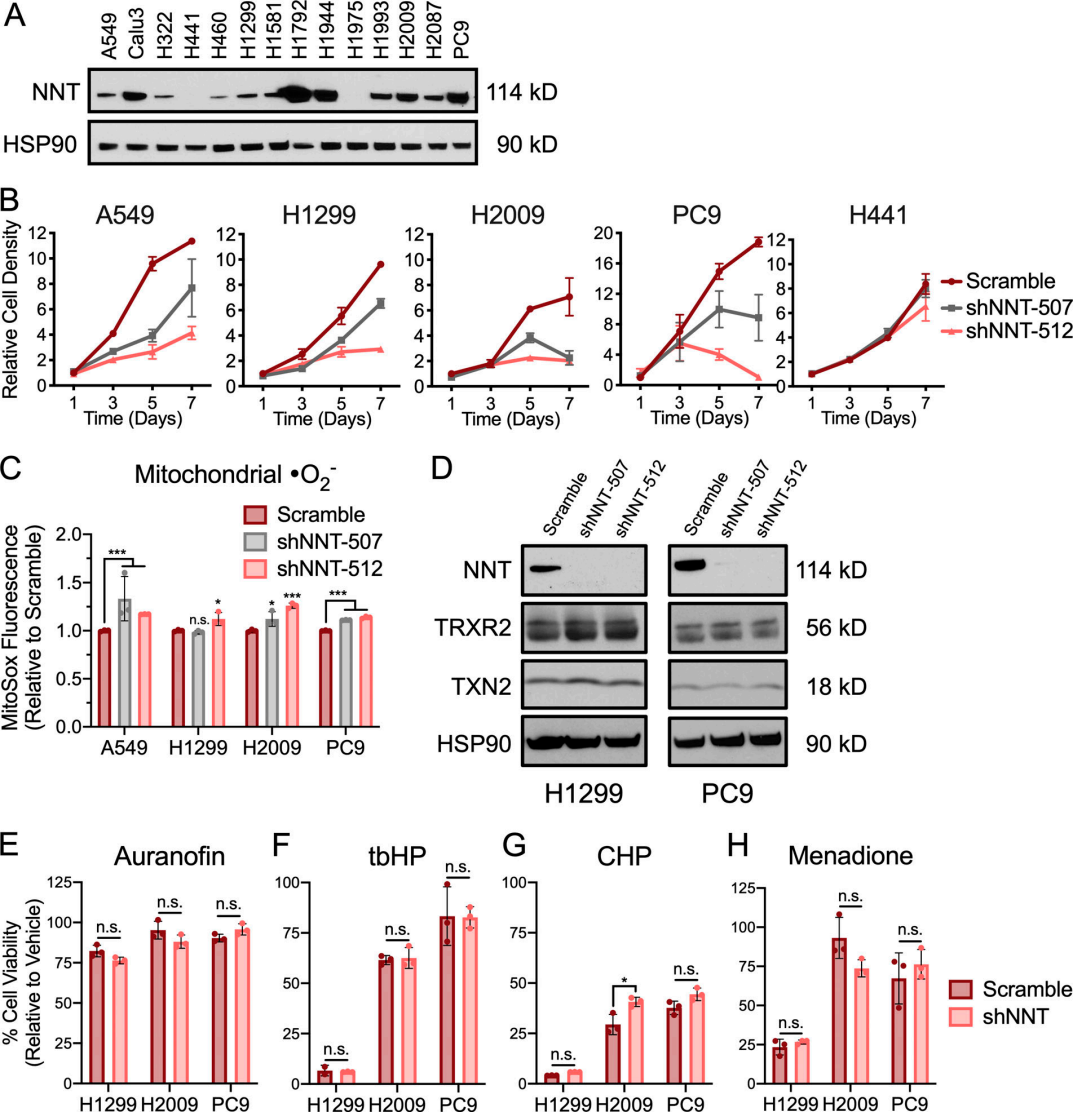
**NNT loss does not sensitize NSCLC cells to exogenous oxidants.** **(A)** Immunoblot analysis of NNT and HSP90 (loading control) expression in a panel of human NSCLC cells. **(B)** Plots of cell proliferation over 7 d for NSCLC cells subject to scramble or shNNT lentiviral infection. **(C)** Fluorescence of the mitochondrial •O_2_^−^ sensitive dye, MitoSOX Red, in NSCLC cells following NNT knockdown, relative to scramble-infected control cells (one-way ANOVA). **(D)** Immunoblot analysis of NNT, TRXR2, TXN2, and HSP90 (loading control) in lysates of NSCLC cells 4 d after lentiviral infection. **(E–H)** Viability of NSCLC cells subject to scramble or shNNT lentiviral infection following 24 h treatment with (E) 1 µM auranofin or 15 µM (F) tbHP, (G) CHP, or (H) menadione (Student’s *t* test). Cell viability was determined relative to vehicle treated controls. Data are representative of one experiment of three experimental replicates. For B, C, and E–H, data are represented as mean ± SD of three technical replicates. n.s., not significant; *, P < 0.05; ***, P < 0.001.

**Figure 2.**
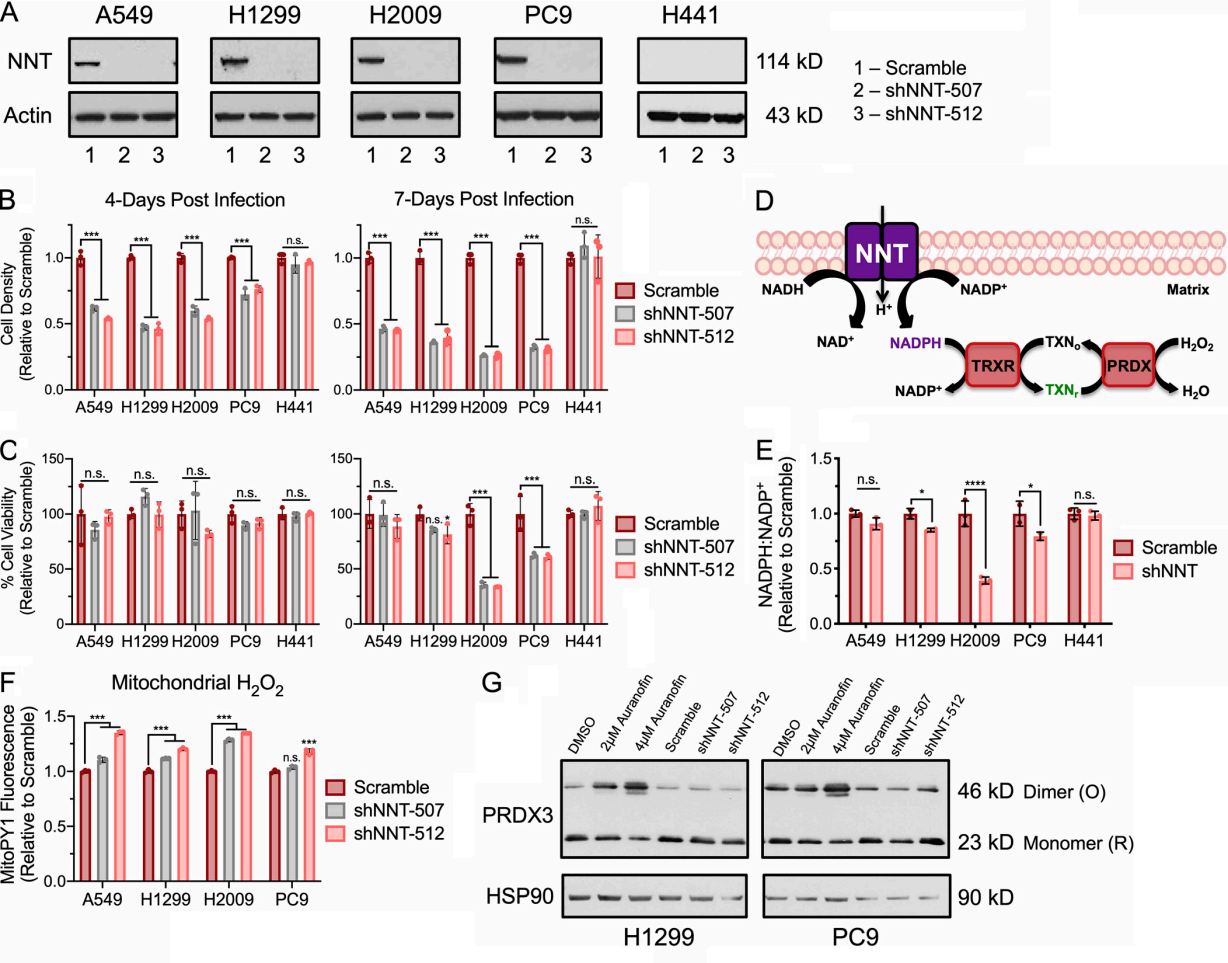
**NNT loss does not compromise the mitochondrial TXN antioxidant system. (A)** Immunoblot analysis of NNT and actin (loading control) expression in NSCLC cells 3 d after infection with scramble or shNNT lentivirus. **(B)** Relative cell density of NSCLC cells 4 or 7 d after infection with scramble or shNNT lentivirus (one-way ANOVA). **(C)** Relative viability of NSCLC cells 4 or 7 d after infection with scramble or shNNT lentivirus (one-way ANOVA). **(D)** Schematic representation of the canonical function of NNT, where NNT supplies NADPH to maintain mitochondrial antioxidant capacity. TXN: oxidized (TXN_o_), reduced (TXN_r_). **(E)** NADPH:NADP^+^ ratio in NSCLC cells following NNT knockdown, relative to scramble infected control cells (Student’s *t* test). **(F)** Fluorescence of the mitochondrial H_2_O_2_ sensitive dye, MitoPY1, in NSCLC cells following NNT knockdown relative to scramble infected control cells (one-way ANOVA). **(G)** Redox immunoblot analysis of HSP90 (loading control) and the oxidation state of PRDX3 in parental NSCLC cells following 4 h treatment with DMSO, 2 µM, or 4 µM auranofin or 4 d after infection with scramble or shNNT lentivirus. Data are representative of one experiment of three experimental replicates. For B–F, data are represented as mean ± SD of three technical replicates. n.s., not significant; *, P < 0.05; ***, P < 0.001; ****, P < 0.0001.

To further evaluate the influence of NNT on lung tumor biology, we transitioned to human NSCLC cell lines, which exhibit varied NNT expression (Fig. S1 A). We first assessed the effect of shRNA-mediated knockdown of NNT on the proliferative capacity of four NSCLC cell lines that express NNT (A549, H1299, H2009, and PC9) as well as H441 cells, which do not express NNT protein and serve as a natural negative control (Fig. 2 A). We observed that shRNA-mediated knockdown of NNT with two unique hairpins blunted the proliferative capacity of NNT-expressing NSCLC cells (Figs. 2 B and S1 B). Further, NNT knockdown compromised the viability of H2009 and PC9 cells beyond 4 d after lentiviral infection (Figs. 2 C and S1 B). Importantly, the proliferation of H441 cells was not affected by NNT knockdown, indicating specificity of the hairpins.

Canonically, NNT is thought to contribute the reducing power (NADPH) required to maintain the mitochondrial protein antioxidant system in a reduced state (Fig. 2 D; Rydström, 2006). We found that NNT knockdown reduced the cellular NADPH:NADP^+^ ratio in several cell lines (H1299, H2009, and PC9) while having no effect on H441 cells (Fig. 2 E). Consistent with the notion that NNT is important for H_2_O_2_ detoxification, we observed modest, yet statistically significant increases in mitochondrial H_2_O_2_ levels 4 d following lentiviral infection (Fig. 2 F). We also observed modest increases in mitochondrial superoxide (•O_2_^−^) following NNT knockdown (Fig. S1 C).

To determine if these increases in mitochondrial ROS related to the mitochondrial antioxidant system, we assessed the oxidation state of the mitochondrial peroxiredoxin (PRDX) 3 through redox Western blotting. H_2_O_2_ detoxification by PRDX3 induces dimerization of the protein through a pair of cysteine disulfide bonds that must be reduced by TXN in order to restore PRDX3 antioxidant function. Thus, accumulation of dimerized PRDX3 is an indication of PRDX3 oxidation and a surrogate marker for mitochondrial oxidative stress (Cox et al., 2009). While we found that treatment with auranofin, an inhibitor of TXN reductase (TRXR), resulted in substantial oxidation of PRDX3, the loss of NNT did not increase PRDX3 oxidation relative to scramble-infected control cells (Fig. 2 G). Further, NNT loss did not decrease protein levels of the mitochondrial TXN2 or TRXR2 (Fig. S1 D). Consistent with this, we found that NNT knockdown did not sensitize NSCLC cells to treatment with auranofin (Fig. S1 E). Moreover, NNT knockdown had no effect on the sensitivity of NSCLC cells to the oxidants tert-butyl hydroperoxide (tbHP), cumene hydroperoxide (CHP), or the mitochondrial-targeted menadione (Fig. S1, F–H).

**Figure S2.**
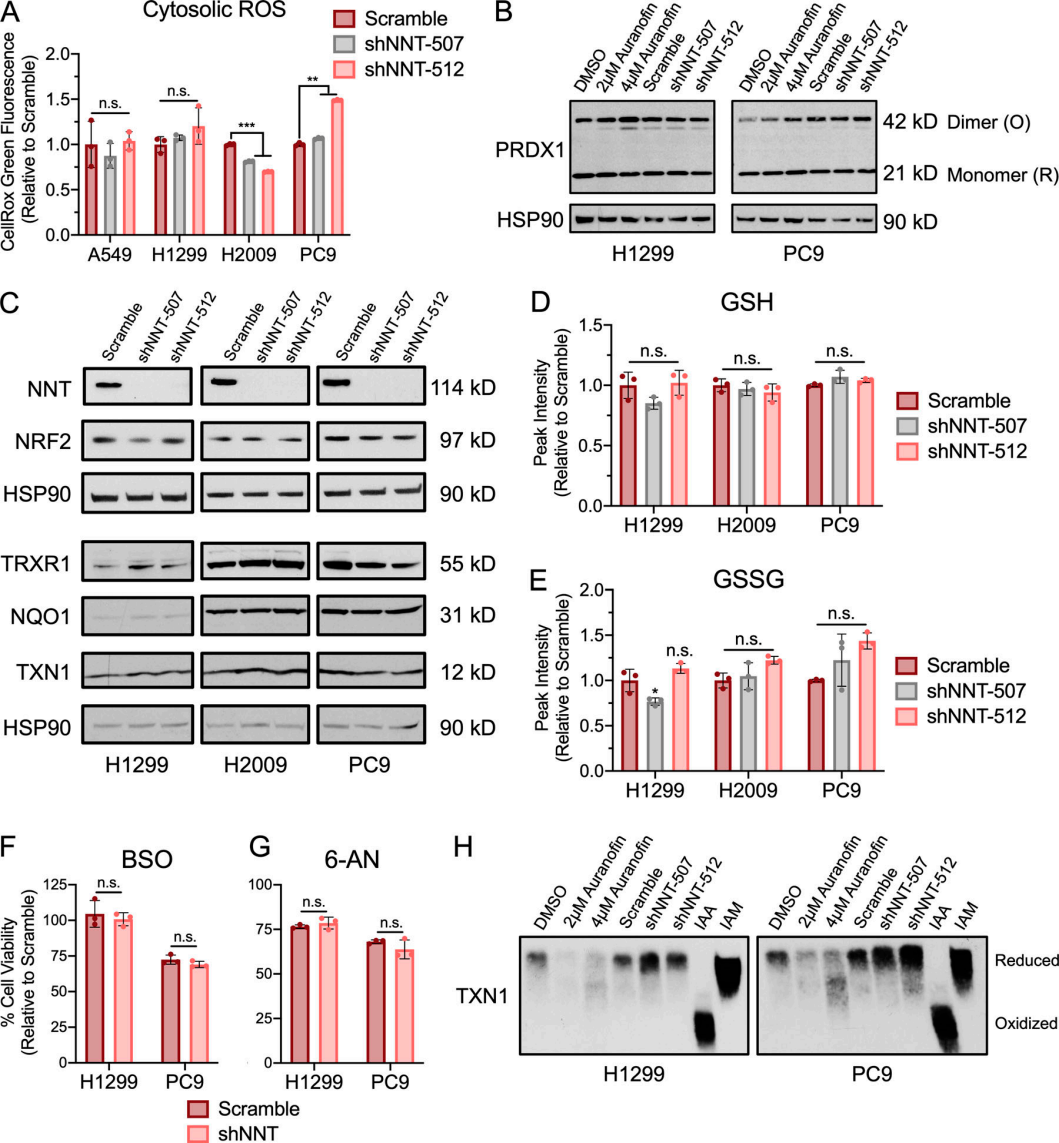
**NNT knockdown does not induce cytosolic oxidative stress.** **(A)** Fluorescence of the cytosolic ROS-sensitive dye, CellROX Green, in NSCLC cells following NNT knockdown, relative to scramble-infected control cells (one-way ANOVA). **(B)** Redox immunoblot analysis of HSP90 (loading control) and the oxidation state of PRDX1 in parental NSCLC cells following 4 h treatment with DMSO, 2 µM, or 4 µM auranofin or 4 d after infection with scramble or shNNT lentivirus. **(C)** Immunoblot analysis of protein expression in lysates of NSCLC 4 d after lentiviral infection; HSP90 (loading control), NNT, NRF2 and its downstream targets TRXR1, NQO1, TXN1. **(D)** Relative abundance of reduced GSH in extracts of NSCLC cells subject to NNT knockdown (one-way ANOVA). **(E)** Relative abundance of oxidized glutathione (GSSG) in extracts of NSCLC cells subject to NNT knockdown (one-way ANOVA). **(F and G)** Viability of NSCLC cells subject to scramble or shNNT lentiviral infection following 24 h treatment with 100 µM (F) BSO or (G) 6-aminonicotinamide (6-AN; Student’s *t* test). **(H)** Redox immunoblot analysis of the oxidation state of TXN1 in parental NSCLC cells following 4 h treatment with DMSO, 2 µM, or 4 µM auranofin or 4 d after infection with scramble or shNNT lentivirus. Control samples derivatized in either iodoacetic acid (IAA) or iodoacetamide (IAM) were included to denote oxidized and reduced TXN1 signals, respectively. For A–C and F–H, data are representative of one experiment of three experimental replicates. For D and E, data are represented as the mean fold change relative to scramble controls of three biological replicates. For A and D–G, data are represented as mean ± SD of three technical or biological replicates. n.s., not significant; *, P < 0.05; **, P < 0.01; ***, P < 0.001.

To determine whether NNT loss elicited extramitochondrial oxidative stress, we evaluated cytosolic ROS levels and found no discernable effect with NNT knockdown (Fig. S2 A). This coincided with a lack of oxidation of the cytosolic peroxiredoxin PRDX1 (Fig. S2 B). Furthermore, we did not observe an induction of nuclear factor erythroid 2–related factor 2 (NRF2), the master regulator of cellular antioxidant capacity, or its downstream targets (Fig. S2 C). Consistent with a lack of overt oxidative stress, NNT knockdown did not alter the levels of glutathione in either its reduced (GSH) or oxidized forms (Fig. S2, D and E). Moreover, NNT loss failed to sensitize NSCLC cells to the GSH synthesis inhibitor, buthionine sulfoximine (BSO; Fig. S2 F). Interestingly, NNT knockdown also had no effect of the sensitivity of NSCLC cells to inhibition of the pentose phosphate pathway, a major source of cytosolic NADPH (Fig. S2 G; Lewis et al., 2014). We also found that the oxidation state of TXN1 did not change with NNT loss (Fig. S2 H). Collectively, these results indicate that NNT is important for the proliferative capacity of NSCLC cells but that loss of NNT activity does not impair the mitochondrial TXN antioxidant system or elicit significant oxidative stress.

### NNT loss compromises mitochondrial oxidative capacity

**Figure 3.**
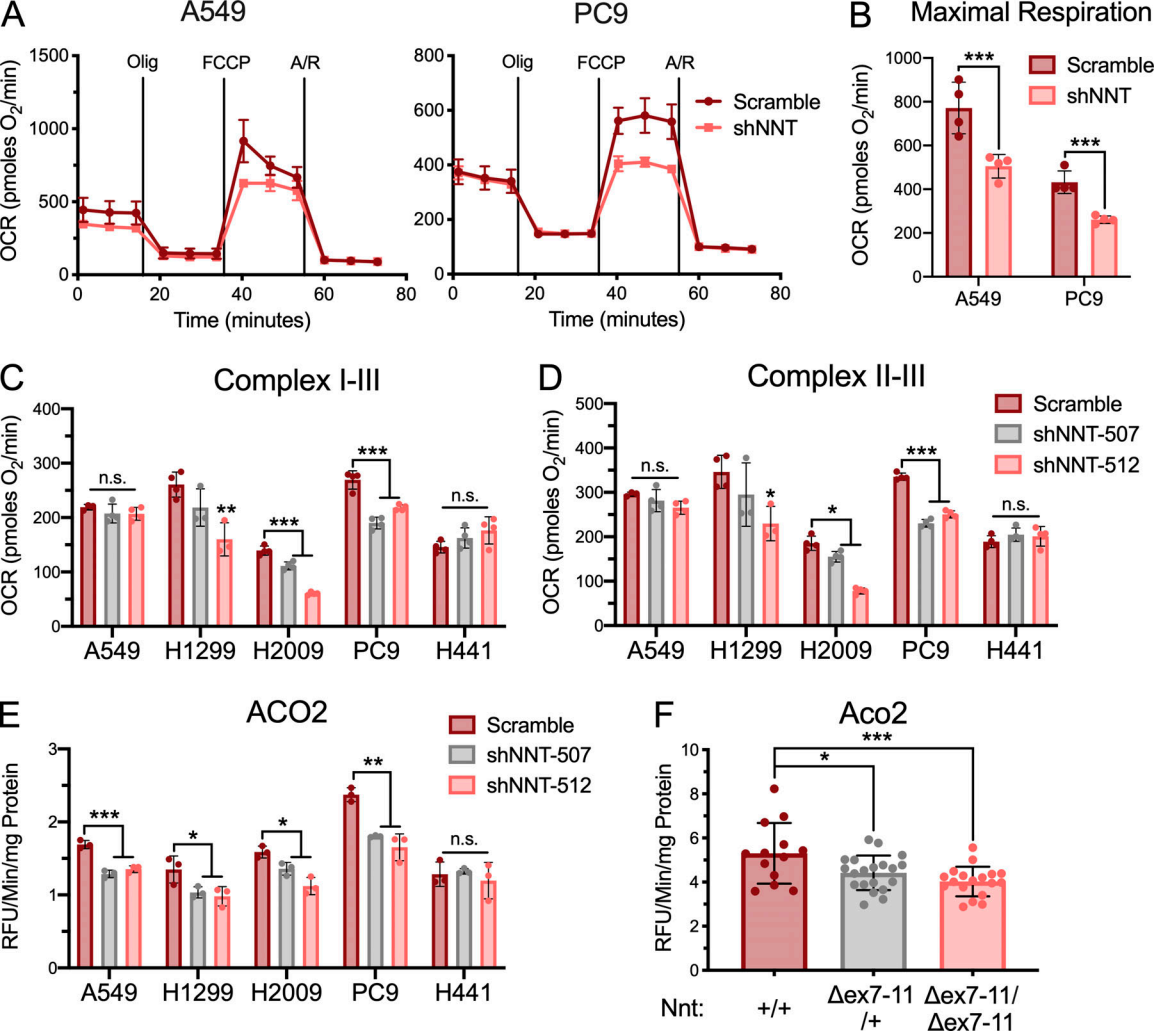
**NNT loss compromises mitochondrial oxidative capacity. (A)** Plots of OCR in A549 and PC9 cells subject to NNT knockdown. Cells were supplemented with 10 mM glucose and 1 mM glutamine and then sequentially challenged with 1 µM oligomycin (Olig), 1 µM (A549) or 0.5 µM (PC9) of FCCP, and 1 µM each of antimycin A (A) and rotenone (R). **(B)** Average maximal respiratory capacity of A549 and PC9 cells following infection with either scramble or shNNT lentivirus (Student’s *t* test). **(C)** Average complex I–III activity following stimulation with 10 mM pyruvate and 1 mM malate in NSCLC cells subject to NNT knockdown (one-way ANOVA). **(D)** Average complex II–III activity following stimulation with 10 mM succinate in NSCLC cells subject to NNT knockdown (one-way ANOVA). **(E)** Average ACO2 activity in mitochondrial lysates of NSCLC cells following NNT knockdown (one-way ANOVA). **(F)** Average ACO2 activity in mitochondrial lysates of lung tumors collected from *LSL-Kras*^*G12D/+*^*; Trp53*^*flox/flox*^*; Nnt*^*+/+*^ (*n* = 13 tumors from five mice), *LSL-Kras*^*G12D/+*^*; Trp53*^*flox/flox*^*; Nnt*^*Δex7-11/+*^ (*n* = 20 tumors from five mice), and *LSL-Kras*^*G12D/+*^*; Trp53*^*flox/flox*^*; Nnt*^*Δex7-11/Δex7-11*^ (*n* = 18 tumors from four mice) mice (one-way ANOVA). For A–E, data are representative of one experiment of three experimental replicates. For A–E, data are represented as mean ± SD of at least three technical replicates. n.s., not significant; *, P < 0.05; **, P < 0.01; ***, P < 0.001. pmoles, picomoles; RFU, relative fluorescence units.

**Figure S3.**
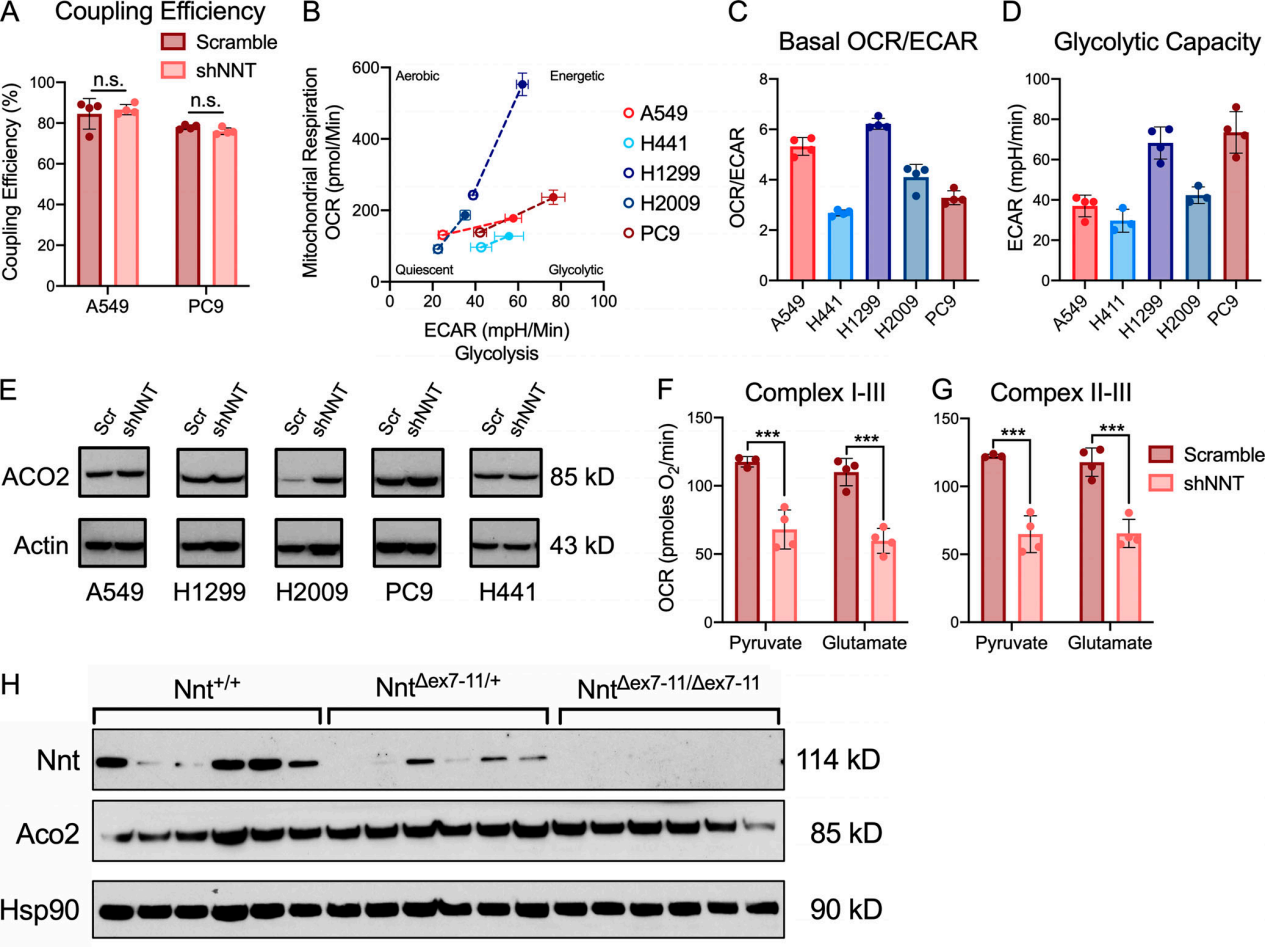
**The effect of NNT loss on ACO2 is independent of changes in protein expression.** **(A)** Average coupling efficiency of A549 and PC9 cells following infection with either scramble or shNNT lentivirus (Student’s *t* test). **(B)** Metabolic phenotyping of NSCLC cells subject to 10 mM glucose and 1 mM glutamine before (open circles) and after cotreatment (closed circles) with oligomycin (1 µM) and FCCP (0.5 µM for H441, H1299, H2009, PC9 cells; 1 µM for A549). Dotted lines are representative of metabolic potential under stress conditions. **(C)** Ratio of basal oxygen consumption (OCR) and extracellular acidification (ECAR) in NSCLC cells subject to 10 mM glucose and 1 mM glutamine. **(D)** ECAR values of NSCLC cells subject to 10 mM glucose following treatment with 1 µM oligomycin. **(E)** Immunoblot analysis of ACO2 and actin (loading control) expression in NSCLC cells subject to NNT knockdown. **(F)** Average complex I–III activity following stimulation with 1 mM malate and either 10 mM pyruvate or 10 mM glutamate in H2009 cells subject to NNT knockdown (one-way ANOVA). **(G)** Average complex II–III activity in H2009 cells subject to NNT knockdown following stimulation with 10 mM succinate in the presence of 1 mM malate and either 10 mM pyruvate or 10 mM glutamate (one-way ANOVA). **(H)** Immunoblot analysis of Nnt, Aco2, and Hsp90 (loading control) expression in lung tumors collected from *LSL-Kras*^*G12D/+*^*; Trp53*^*flox/flox*^*; Nnt*^*+/+*^ (*n* = 6), *LSL-Kras*^*G12D/+*^*; Trp53*^*flox/flox*^*; Nnt*^*Δex7-11/+*^ (*n* = 6), and *LSL-Kras*^*G12D/+*^*; Trp53*^*flox/flox*^*; Nnt*^*Δex7-11/Δex7-11*^ (*n* = 6) mice. Data are representative of one experiment of three experimental replicates. For A–D, F, and G, data are represented as mean ± SD of at least three technical replicates. n.s., not significant; ***, P < 0.001.

Given the localization of NNT within the IMM and its ability to influence proton flux across the membrane and reducing power, we sought to interrogate whether NNT was important for mitochondrial oxidative metabolism. First, we employed the seahorse extracellular flux analyzer to perform a MitoStress test as an assessment of the impact of NNT loss on general mitochondrial oxidative function. We observed that the oxygen consumption rate (OCR) of NNT-deficient cells was reduced relative to scramble control cells in response to the sequential delivery of mitochondrial inhibitors (Fig. 3 A). Notably, the maximal respiratory capacity of NNT-deficient cells was significantly lower independent of an effect on uncoupled respiration (Figs. 3 B and S3 A). This is indicative of a mitochondrial oxidative defect that is unrelated to NNT’s influence on the proton gradient. Given the significant effect of NNT loss on respiratory capacity, we examined whether the NNT-deficient H441 cells were inherently less oxidative and more glycolytic than NNT-expressing cells. Basal metabolic phenotyping revealed that H441 cells exhibit a basal OCR/extracellular acidification rate (ECAR) ratio similar to that of other lines evaluated here and do not exhibit enhanced glycolytic capacity (Fig. S3, B–D). This suggests that these cells have evolved to maintain mitochondrial function even in the absence of NNT, likely through engagement of alternative sources of mitochondrial NADPH.

Mitochondrial oxidative metabolism is dependent on a functional ETC, which consists of protein complexes with resident Fe-S clusters that mediate electron transport. Considering that NADPH is required for Fe-S cluster biosynthesis, we endeavored to examine if the decrease in mitochondrial respiratory function following NNT knockdown was linked to the Fe-S proteins within the ETC (Webert et al., 2014). While the MitoStress test allows for a general analysis of respiratory function, it does not allow for the evaluation of individual ETC complexes. Therefore, we performed a more specialized seahorse-based protocol that permits the sequential analysis of the Fe-S cluster-dependent respiratory complexes (I, II, and III; Salabei et al., 2014). We found that in response to feeding with the complex I substrates, pyruvate and malate, the activity of complex I–III was significantly reduced following NNT knockdown (Fig. 3 C). Furthermore, NNT-deficient cells also exhibited significantly reduced OCR in response to succinate, indicative of reduced succinate dehydrogenase (SDH) activity and flux through complex II–III (Fig. 3 D). This is particularly consequential as SDH serves a dual role in the ETC and TCA cycle. As expected, NNT knockdown did not alter the Fe-S cluster-dependent respiratory chain activities of H441 cells (Fig. 3, C and D).

In addition to sustaining electron flux through the ETC, Fe-S clusters support the enzymatic function of other proteins critical to oxidative metabolism. To determine if NNT contributes to the function of other Fe-S proteins, we assessed the activity of aconitase (ACO2), a Fe-S protein of the TCA cycle. We found that NNT knockdown significantly reduced ACO2 activity in those NSCLC lines with NNT expression (Fig. 3 E). This reduction in ACO2 activity occurred independently from a decrease in ACO2 expression, suggesting that the change in activity was due to a functional deficit (Fig. S3 E).

Diminished ACO2 activity is likely to disrupt TCA cycling, leading to a reduced capacity to generate the reducing equivalents needed to drive ETC flux. To ensure that the decreased respiratory chain phenotypes we observed were not simply consequences of this ACO2 defect, we assessed respiratory chain function in response to glutamate and malate stimulation. Glutamate carbon can enter the TCA cycle as α-ketoglutarate, permitting us to circumvent the need for ACO2, which is required for the initial turn of pyruvate carbon through the cycle. Regardless, NNT-deficient cells exhibited equally disrupted respiratory chain function in response to glutamate as they did to pyruvate (Fig. S3, F and G). To supplement the analyses of ACO2 function in out NSCLC cell lines, we also evaluated the influence of Nnt expression on Aco2 activity in KP lung tumors. We found that Aco2 activity was significantly higher in tumors from *Nnt*^*+/+*^ mice than those of *Nnt*^*Δex7-11/+*^ and *Nnt*^*Δex7-11/Δex7-11*^, with tumors lacking Nnt exhibiting the lowest activity (Fig. 3 F). While Nnt expression varied between tumors of the same genotype, it was expectedly higher in *Nnt*^*+/+*^ tumors relative to *Nnt*^*Δex7-11/+*^ tumors and completely absent in *Nnt*^*Δex7-11/Δex7-11*^ tumors (Fig. S3 H). Furthermore, as in the human cell lines, the difference in Aco2 activity was not the result of differential protein expression (Fig. S3 H).

### An exogenous source of NADPH sustains Fe-S protein function following NNT loss

**Figure 4.**
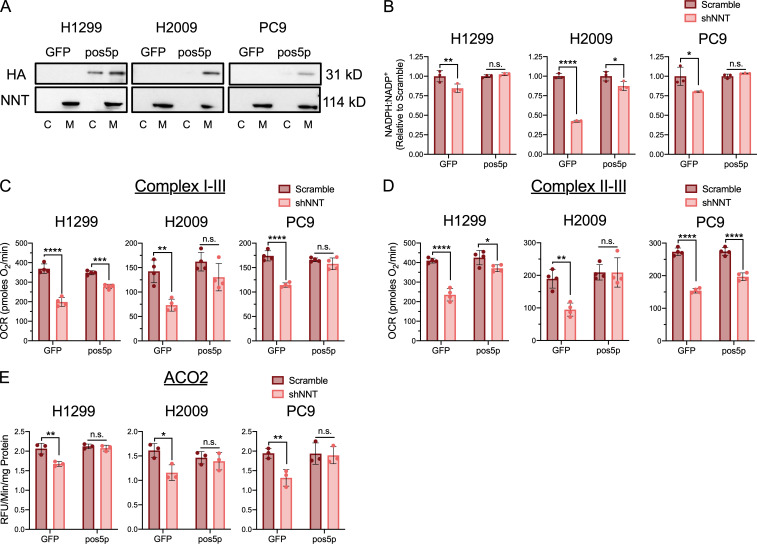
**An exogenous source of NADPH sustains Fe-S protein function following NNT loss. (A)** Immunoblot analysis of HA-tag and NNT (mitochondrial control) expression in cytosolic (C) and mitochondrial (M) lysates taken from GFP or pos5p expressing NSCLC cells. **(B)** NADPH:NADP^+^ ratio in GFP or pos5p-expressing NSCLC cells following NNT knockdown, relative to scramble infected control cells (two-way ANOVA). **(C)** Average complex I–III activity following stimulation with 10 mM pyruvate and 1 mM malate in GFP or pos5p-expressing NSCLC cells subject to NNT knockdown (two-way ANOVA). **(D)** Average complex II–III activity following stimulation with 10 mM succinate in GFP or pos5p-expressing NSCLC cells subject to NNT knockdown (two-way ANOVA). **(E)** Average ACO2 activity in mitochondrial lysates of GFP or pos5p expressing NSCLC cells following NNT knockdown (two-way ANOVA). Data are representative of one experiment of three experimental replicates. For B–E, data are represented as mean ± SD of at least three technical replicates. n.s., not significant; *, P < 0.05; **, P < 0.01; ***, P < 0.001; ****, P < 0.0001.

**Figure S4.**
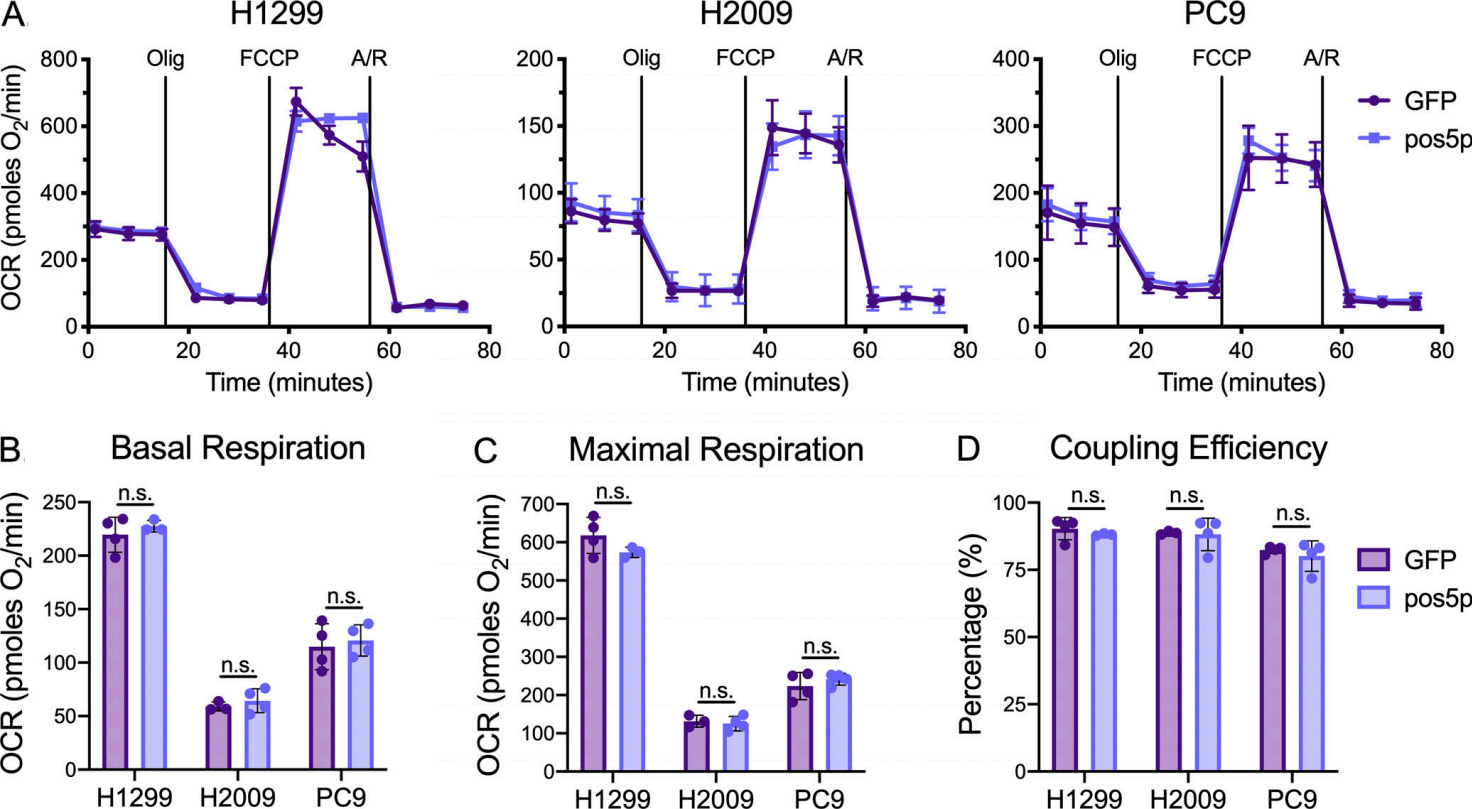
**Pos5p expression does not disrupt mitochondrial function in NSCLC cells.** **(A)** Plots of OCR in GFP or pos5p-expressing NSCLC cells. Cells were supplemented with 10 mM glucose and 1 mM glutamine and then sequentially challenged with 1 µM oligomycin (Olig), 0.5 µM of FCCP, and 1 µM each of antimycin A (A) and rotenone (R). **(B–D)** Average measures of (B) basal respiration, (C) maximal respiration, and (D) coupling efficiency in GFP or pos5p-expressing NSCLC cells (Student’s *t* test). Data are representative of one experiment of three experimental replicates. Data are represented as mean ± SD of at least three technical replicates. n.s., not significant.

To determine if the decreases in mitochondrial Fe-S protein function associated with NNT knockdown were related to the accompanying decrease in NADPH:NADP^+^, we sought to provide an exogenous source of mitochondrial NADPH. To achieve this, we chose the yeast mitochondrial NADH kinase, pos5p, which phosphorylates NADH to yield NADPH (Pain et al., 2010). Though pos5p has been exogenously expressed in bacteria previously (Lee et al., 2013), to our knowledge it has not been introduced into a mammalian system. To monitor if we could efficiently express pos5p protein in the mitochondria of our human NSCLC cells, we modified the yeast protein to include an HA-tag. Western blot analysis of fractionated lysates revealed successful expression of HA-tagged pos5p in the mitochondria of H1299, H2009, and PC9 cells (Fig. 4 A). These lines were chosen to evaluate the ability of pos5p to rescue the Fe-S protein defects associated with NNT loss as they exhibited the most severe response to NNT knockdown. Importantly, we did not observe any adverse effects of pos5p expression on mitochondrial function in our NSCLC cells (Fig. S4).

Expression of pos5p rescued the decrease in the cellular NADPH:NADP^+^ ratio elicited by NNT knockdown (Fig. 4 B). This corresponded with an attenuation of the decrease in respiratory chain complex activities following knockdown of those cells expressing pos5p (Fig. 4, C and D). Moreover, pos5p expression fully rescued the decrease in ACO2 activity associated with NNT knockdown (Fig. 4 E). Together, these data indicate that maintaining NADPH levels upon loss of NNT expression protects Fe-S protein function in NSCLC cells.

### NNT loss does not disrupt Fe-S cluster biosynthesis

Given that an exogenous source of mitochondrial NADPH attenuated the Fe-S protein defects associated with NNT knockdown and that NADPH is required for efficient and sustained Fe-S cluster biosynthesis, we next sought to determine if NNT activity sustained this process. Fe-S cluster biosynthesis occurs at a multiprotein complex consisting in part of the cysteine desulfurase (NFS1) and Fe-S scaffold protein (ISCU; Johnson et al., 2005). Loss of either compromises cluster biosynthesis and is associated with mitochondrial defects (Fosset et al., 2006; Crooks et al., 2018). Therefore, we introduced shRNAs targeting either NFS1 or ISCU (Alvarez et al., 2017) to establish the effects of disrupting Fe-S cluster biosynthesis on the respiratory chain and ACO2.

**Figure 5.**
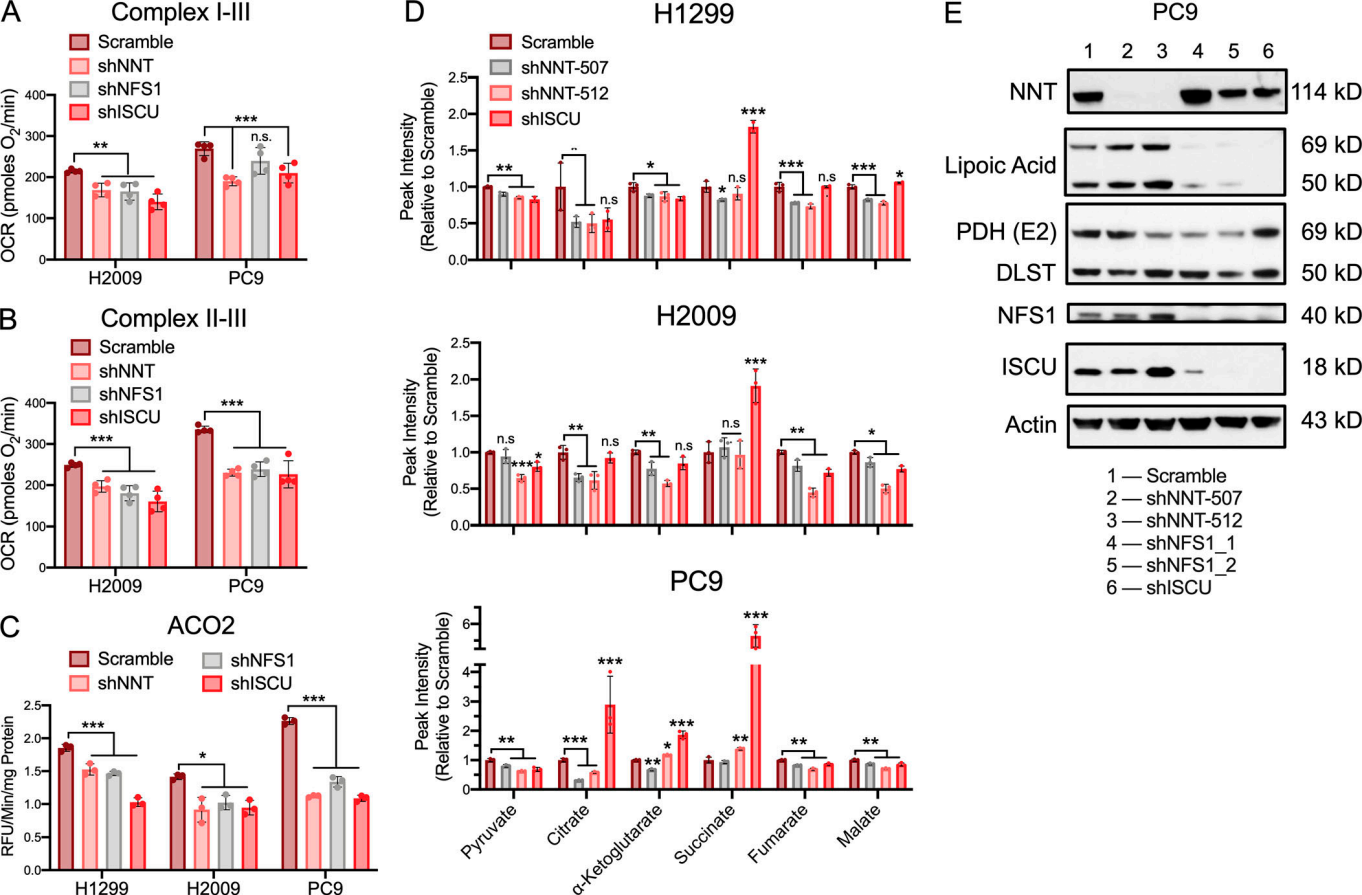
**NNT loss does not disrupt Fe-S cluster biosynthesis. (A)** Average complex I–III activity following stimulation with 10 mM pyruvate and 1 mM malate in H2009 and PC9 cells subject to NNT, NFS1, or ISCU knockdown (one-way ANOVA). **(B)** Average complex II–III activity following stimulation with 10 mM pyruvate and 1 mM malate in H2009 and PC9 cells subject to NNT, NFS1, or ISCU knockdown (one-way ANOVA). **(C)** Average ACO2 activity in mitochondrial lysates of NSCLC cells following NNT, NFS1, or ISCU knockdown (one-way ANOVA). **(D)** Relative abundance of TCA cycle intermediates in extracts of NSCLC cells subject to NNT or ISCU knockdown (one-way ANOVA). **(E)** Immunoblot analysis of NNT, lipoic acid, PDH-E2, DLST, NFS1, ISCU, and actin (loading control) expression in PC9 cells following infection with (1) scramble, (2) shNNT-507, (3) shNNT-512, (4) shNFS1_1, (5) shNFS1_2, or (6) ISCU lentivirus. For A–C, data are representative of one experiment of three experimental replicates. For A–C, data are represented as mean ± SD of at least three technical replicates. For D, data are represented as mean ± SD of three biological replicates. n.s., not significant; *, P < 0.05; **, P < 0.01; ***, P < 0.001.

NFS1 knockdown significantly diminished the activities of the respiratory chain complexes in response to both pyruvate/malate as well as succinate in H2009 cells, whereas only complex II–III activity was significantly reduced in PC9 cells (Fig. 5, A and B). Alternatively, loss of ISCU expression significantly blunted OCR in response to pyruvate/malate and succinate in both cell lines (Fig. 5, A and B). Furthermore, knockdown of either NFS1 or ISCU significantly reduced ACO2 activity across cell lines (Fig. 5 C). Intriguingly, the deficits elicited by NNT knockdown were of equal magnitude to those resultant from targeting these bona fide components of the Fe-S cluster biosynthetic machinery (Fig. 5, A–C).

To demonstrate that the Fe-S protein defects we observed have a functional impact on the mitochondrial metabolism of these NSCLC cells, we performed liquid chromatography-high resolution mass spectrometry (LC-HRMS)–based metabolomics on cells subjected to NNT or ISCU knockdown. Analysis of TCA cycle metabolites from these cells revealed significant alterations in the abundance of most intermediates across cell lines (Fig. 5 D). These are indicative of a severe disruption of oxidative metabolism and consistent with the described defects in Fe-S protein function. Specifically, we observed a depletion of pyruvate, malate, and fumarate following disruption of both NNT and ISCU expression. While citrate levels were depleted in NNT-deficient cells, there was no consistent effect of ISCU knockdown. Alternatively, ISCU-deficient cells exhibited a striking accumulation of succinate that was absent in NNT knockdown cells (Fig. 5 D).

In addition to the Fe-S proteins ACO2 and SDH, TCA cycling is dependent on the function of an additional mitochondrial Fe-S protein, lipoic acid synthetase (LIAS). LIAS is required for lipoic acid synthesis and the eventual conjugation of crucial lipoate moieties to components of PDH (E2) and α-ketoglutarate dehydrogenase (dihydrolipoamide S-succinyltransferase, DLST), among others. LIAS is critically sensitive to disruptions in Fe-S cluster biosynthesis, as its resident Fe-S cluster is consumed during catalysis, imposing a requirement for continual cluster turnover (Crooks et al., 2018). Indeed, disruption of NFS1 and ISCU expression resulted in a substantial reduction in PDH-E2 and DLST lipoylation in PC9 cells (Fig. 5 E). However, NNT knockdown had no effect on protein lipoylation (Fig. 5 E). Collectively, these data suggest that while NNT elicits enzymatic and metabolic defects reminiscent of those associated with the disruption of Fe-S cluster biosynthesis, it is unlikely that NNT directly influences this process. This is reflected in the similar yet distinct effects of NNT and ISCU knockdown on TCA cycle intermediate levels.

### NNT loss disrupts fatty acid metabolism

**Figure 6.**
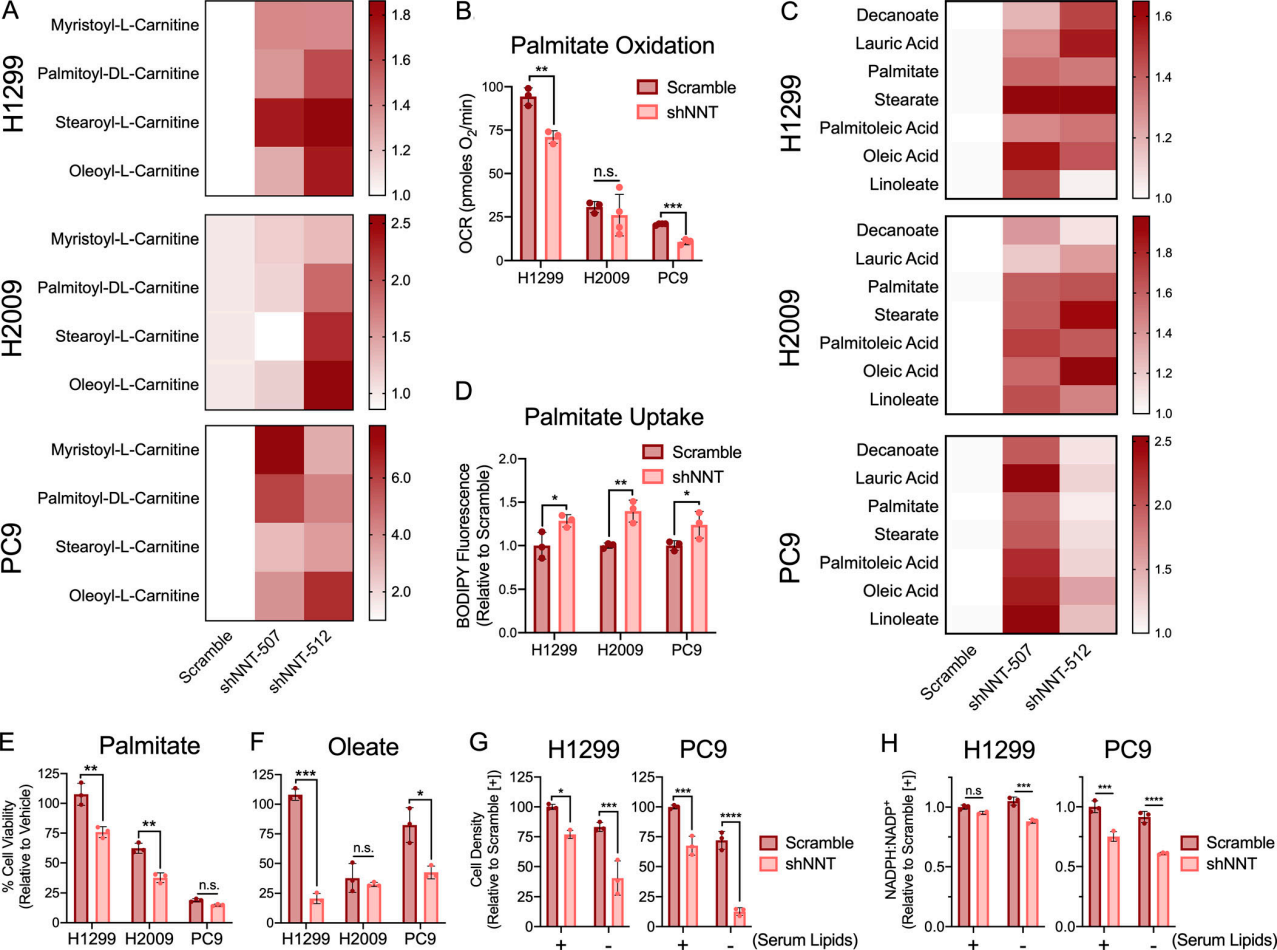
**NNT loss disrupts fatty acid metabolism. (A)** Heat map representation of the relative abundances of long chain fatty acyl-carnitines in extracts of NSCLC cells following NNT knockdown. **(B)** Measures of OCR coupled to the oxidation of exogenous palmitate-BSA in NSCLC cells subject to NNT knockdown (Student’s *t* test). **(C)** Heat map representation of the relative abundances of saturated (decanoate, lauric acid, palmitate, stearate) and unsaturated (palmitoleic acid, elaidic acid, linoleate) fatty acids in extracts of NSCLC cells following NNT knockdown. **(D)** Relative BODIPY-FL C16 fluorescence of scramble or shNNT-infected NSCLC cells following a 30-min incubation with the fluorescent palmitate analogue (Student’s *t* test). **(E and F)** Viability of NSCLC cells subject to scramble or shNNT lentiviral infection following 24 h treatment with (E) 200 µM of palmitate or (F) 400 µM of oleate (Student’s *t* test). Cell viability was determined relative to BSA treated controls. **(G)** Viability of NSCLC cells subject to scramble or shNNT lentiviral infection following a 48-h incubation in cell culture media with or without serum lipids. Cell viability was determined relative to scramble controls treated with lipid replete media. **(H)** NADPH:NADP^+^ ratio in NSCLC cells subject to scramble or shNNT lentiviral infection following a 24-h incubation in cell culture media with or without serum lipids. Ratio normalized to scramble controls incubated in lipid replete conditions (two-way ANOVA). For A and C, data are represented as the mean fold increase relative to scramble controls of three biological replicates. For B, D, and E–H, data are representative of one experiment of three experimental replicates. For B, D, and E–H, data are represented as mean ± SD of three technical replicates. n.s., not significant; *, P < 0.05; **, P < 0.01; ***, P < 0.001; ****, P < 0.0001.

In addition to the depletion of TCA cycle intermediates, LC-HRMS analysis of NNT-deficient cells revealed a metabolic signature indicative of dysregulated fatty acid metabolism. NNT knockdown promoted the significant accumulation of long chain fatty acyl-carnitines, which serve as substrates for β-oxidation (Fig. 6 A). Given the established respiratory defects seen in NNT-deficient cells, we anticipated that the increase in these acyl-carnitines was a result of decreased fatty acid oxidation. Consistently, we found that OCR linked to palmitate oxidation was reduced in H1299 and PC9 cells following NNT knockdown (Fig. 6 B). We also observed an accumulation of both saturated and unsaturated fatty acids in NNT-deficient cells relative to scramble-infected controls (Fig. 6 C). Given that fatty acid synthesis is highly NADPH-consuming (Fan et al., 2014; Hosios and Vander Heiden, 2018) and NADPH availability was reduced upon NNT loss, we hypothesized that these increases in fatty acid levels were resultant from increased uptake of exogenous fatty acids. Indeed, we found that NNT-deficient cells exhibited an increased capacity to take up a fluorescent palmitate analogue (Fig. 6 D). To evaluate if the accumulation of fatty acids following NNT knockdown is a potential liability, we challenged NNT-deficient cells with the saturated fatty acid palmitate for 24 h. We found that NNT knockdown sensitized H1299 and H2009 cells to palmitate treatment (Fig. 6 E). Furthermore, NNT knockdown significantly sensitized H1299 and PC9 cells to treatment with the monounsaturated fatty acid oleate (Fig. 6 F). Given the deleterious effects of exogenous fatty acid supplementation, we anticipated that depletion of extracellular lipids would protect against NNT loss. Surprisingly, lipid depletion from the culture media exacerbated the effect of NNT knockdown (Fig. 6 G). Importantly, starvation of extracellular lipids enhanced NADPH depletion following NNT knockdown (Fig. 6 H), suggesting NNT-deficient cells have a reduced capacity to buffer NADPH availability when forced to synthesize fatty acids in the absence of an exogenous source. Collectively, these data suggest that NNT may play a role in regulating fatty acid metabolism and that the perturbation of this metabolism in NSCLC cells may serve as an exploitable vulnerability.

### Mitochondrially targeted catalase rescues Fe-S protein function following NNT loss

**Figure 7.**
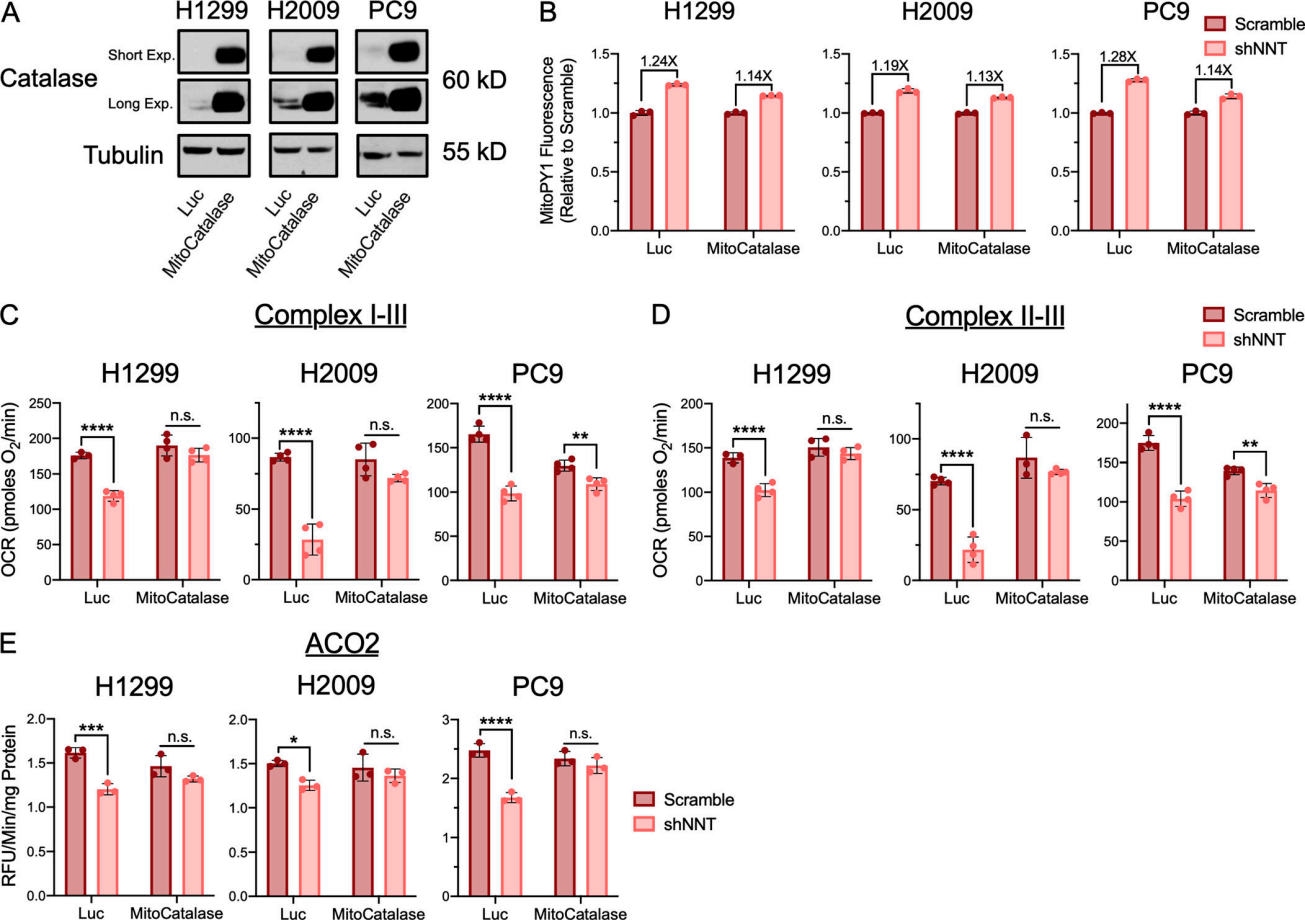
**Mitochondrially targeted catalase rescues Fe-S protein function following NNT loss. (A)** Immunoblot analysis of catalase and tubulin (loading control) expression in Luc or MitoCatalase expressing NSCLC cells. **(B)** Fold inductions of MitoPY1 fluorescence in shNNT-infected Luc or MitoCatalase-expressing NSCLC cells. **(C)** Average complex I–III activity following stimulation with 10 mM pyruvate and 1 mM malate in Luc or MitoCatalase-expressing NSCLC cells subject to NNT knockdown (two-way ANOVA). **(D)** Average complex II–III activity following stimulation with 10 mM succinate in Luc or MitoCatalase-expressing NSCLC cells subject to NNT knockdown (two-way ANOVA). **(E)** Average ACO2 activity in mitochondrial lysates of Luc or MitoCatalase-expressing NSCLC cells following NNT knockdown (two-way ANOVA). Data are representative of one experiment of three experimental replicates. For B–E, data are represented as mean ± SD of at least three technical replicates. n.s., not significant; *, P < 0.05; **, P < 0.01; ***, P < 0.001; ****, P < 0.0001.

**Figure S5.**
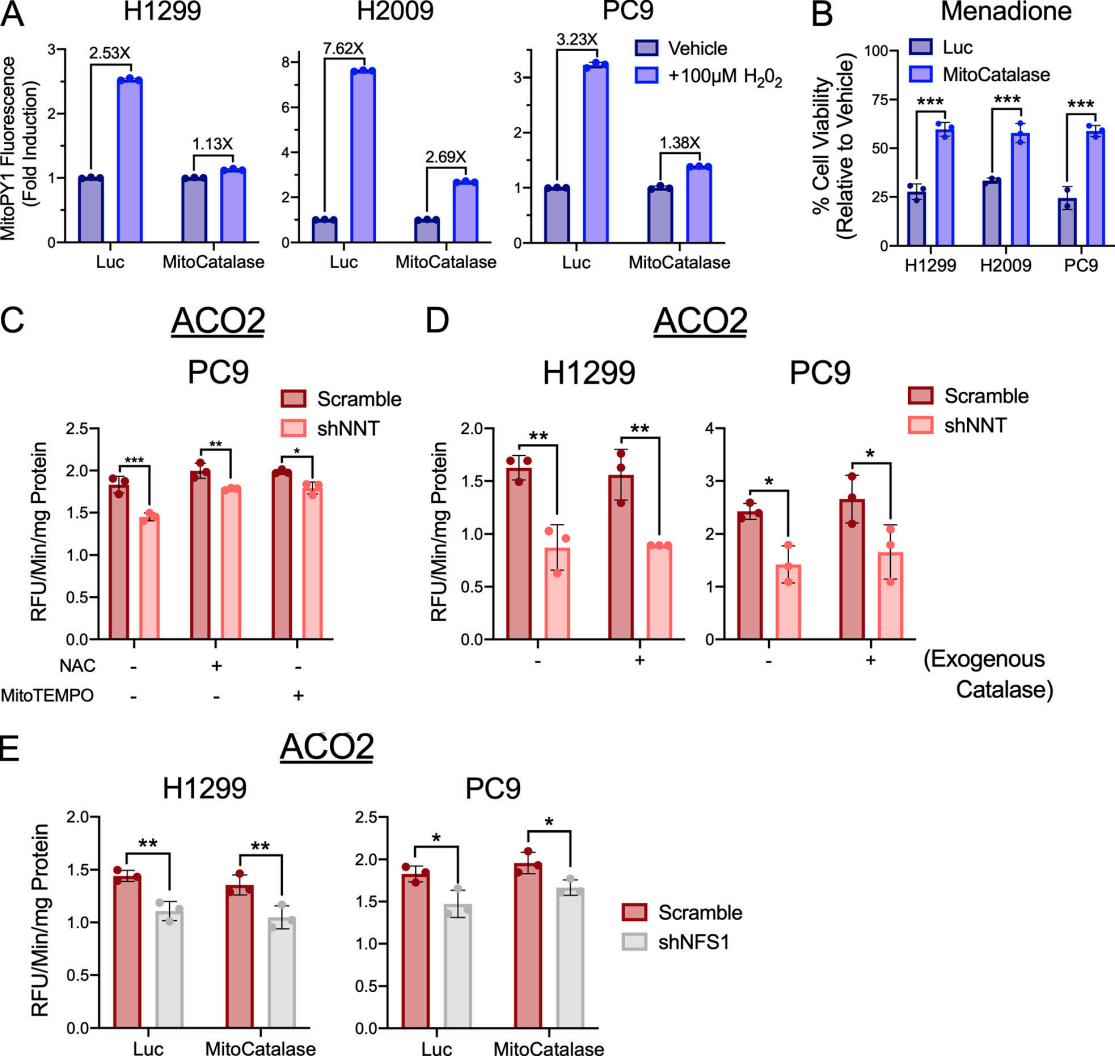
**MitoCatalase protects against mitochondrial oxidation.** **(A)** Fold inductions of MitoPY1 fluorescence in Luc or MitoCatalase-expressing NSCLC cells challenged with 100 µM H_2_O_2_. **(B)** Viability of Luc or MitoCatalase expressing NSCLC cells following 24 h treatment with 15 µM (H1299) or 25 µM (H2009, PC9) menadione (Student’s *t* test). Cell viability was determined relative to vehicle-treated controls. **(C)** Average ACO2 activity in mitochondrial lysates of PC9 cells subject to scramble or shNNT lentiviral infection following a 24-h treatment with vehicle (0.05% DMSO), 1 mM NAC, or 25 nM MitoTEMPO (two-way ANOVA). **(D)** Average ACO2 activity in mitochondrial lysates of NSCLC cells subject to scramble or shNNT lentiviral infection following an overnight treatment with 1,000 U of recombinant catalase (two-way ANOVA). **(E)** Average ACO2 activity in mitochondrial lysates of Luc or MitoCatalase-expressing NSCLC cells following NFS1 knockdown (two-way ANOVA). Data are representative of one experiment of three experimental replicates. Data are represented as mean ± SD of three technical replicates. *, P < 0.05; **, P < 0.01; ***, P < 0.001.

Fe-S clusters are exquisitely sensitive to oxidation by molecular oxygen and more deleterious species (Flint et al., 1993; Djaman et al., 2004; Alvarez et al., 2017). Though we did not observe changes in the oxidation state of the mitochondrial protein antioxidant system, that does not preclude that the modest induction of mitochondrial ROS following NNT knockdown is sufficient to oxidize these sensitive cofactors. To interrogate this possibility, we employed a mitochondrially targeted catalase (MitoCatalase) to enhance mitochondrial antioxidant capacity. We successfully overexpressed MitoCatalase in H1299, H2009, and PC9 cells (Fig. 7 A). These cells exhibited an enhanced capacity to clear mitochondrial H_2_O_2_ upon challenge with 100 µM of exogenous H_2_O_2_ (Fig. S5 A). Furthermore, these MitoCatalase-expressing cells were more resistant to menadione treatment, indicating functionality within the mitochondria (Fig. S5 B).

The expression of MitoCatalase also partially attenuated the modest induction of mitochondrial H_2_O_2_ associated with NNT knockdown (Fig. 7 B). This corresponded with an attenuation of the reduction in respiratory chain complex activities following NNT knockdown (Fig. 7, C and D). Further, MitoCatalase expression rescued ACO2 activity in response to NNT knockdown (Fig. 7 E). This targeted depletion of H_2_O_2_ was recapitulated by the antioxidants N-acetylcysteine (NAC) and the mitochondrial •O_2_^−^ scavenger MitoTempo (Fig. S5 C), which have both been shown to scavenge mitochondrial ROS (Nazarewicz et al., 2013; Ezerina et al., 2018). In contrast, supplementation with untargeted exogenous catalase failed to rescue the effects of NNT knockdown on ACO2 activity, highlighting the need for targeted expression (Fig. S5 D). The MitoCatalase rescue of ACO2 activity was specific to NNT, as NFS1-deficient cells exhibited reduced ACO2 activity even in the presence of MitoCatalase (Fig. S5 E). Collectively, these data indicate that enhancing the mitochondrial capacity to detoxify H_2_O_2_ protects against the Fe-S protein deficits associated with NNT knockdown, further supporting a role for NNT in protecting against Fe-S oxidation, rather than supporting Fe-S cluster biosynthesis.

## Discussion

We demonstrate here for the first time that Nnt expression supports lung tumorigenesis in two genetically engineered mouse models (GEMM) of NSCLC. These GEMMs were crossed with the natural deletion variant of Nnt from a C57BL/6J background (Toye et al., 2005). Intriguingly, C57BL/6J mice are largely resistant to tumor formation (Law et al., 1967), which, in light of our findings, suggests a potential role for Nnt deficiency in this phenotype. Our observation that Nnt expression significantly enhances Kras^G12D^-driven lung tumor formation (Fig. 1, A and B) is consistent with previous work with this GEMM, demonstrating a need for proteins involved in mitochondrial redox metabolism (Weinberg et al., 2010; DeNicola et al., 2011; Davidson et al., 2016; Mayers et al., 2016; Padanad et al., 2016; Rao et al., 2019). A requirement for mitochondrial function in this model aligns with the robust mitochondrial metabolism exhibited by human lung tumors (Hensley et al., 2016). Given this strong evidence for the importance of mitochondrial function in Kras^G12D^-mediated tumorigenesis, it is interesting that the defect in tumor initiation in Nnt-deficient mice can be rescued by p53 loss (Fig. 1, F–H). Loss of p53 function is known to promote glycolytic flux at the expense of mitochondrial metabolism (Zhang et al., 2013; Puzio-Kuter, 2011). While this enhanced Warburg metabolism may permit tumor formation in the absence of optimal mitochondrial function, these tumors appear restricted in their aggressiveness in the absence of Nnt (Fig. 1, I and J). This may be linked to the metabolic stress associated with tumor growth, which dictates metabolic fuel flexibility in response to nutrient deprivation and is diminished with the loss of p53 (Sinthupibulyakit et al., 2010). Nnt expression in these lung tumors may compensate for the lack of p53 support of mitochondrial function (Matoba et al., 2006; Assaily et al., 2011), permitting progression to more advanced stages. In particular, our results (Fig. 6) indicate that Nnt activity may support the capacity of Ras-transformed tumors to use exogenous fatty acids to drive growth under metabolic stress (Kamphorst et al., 2013).

The oxidation of carbon within the mitochondria stimulates ETC flux, which precipitates the formation of •O_2_^−^ and its subsequent conversion to other ROS. This necessitates a functional antioxidant system to prevent an unsustainable accumulation of macromolecular oxidation that can compromise ETC function and mitochondrial integrity. Indeed, activation of NRF2 is a common feature of NSCLC (Singh et al., 2006) and enhances lung tumorigenesis (DeNicola et al., 2011; Romero et al., 2017). Yet in order to sustain this increased antioxidant capacity, cells must maintain a reduced NADPH:NADP^+^ ratio, especially in the mitochondria (Klingenberg and Slenczka, 1959). Given the established function of NNT in sustaining mitochondrial antioxidant capacity through regulation of the NADPH:NADP^+^ ratio (Lopert and Patel, 2014; Ronchi et al., 2016), we anticipated that NNT would serve a similar role in contributing to mitochondrial function in NSCLC. Surprisingly, while we did determine that NNT contributes to the NADPH:NADP^+^ ratio in NSCLC cells, the accompanying increase in mitochondrial ROS was not sufficient to compromise the mitochondrial protein antioxidant system, nor sensitize NNT-deficient cells to treatment with oxidants (Figs. 2 and S1). This is in direct contrast to what was observed in a model of adrenal adenocarcinoma (Chortis et al., 2018), though it was previously established that NNT regulation of global oxidation is critical to adrenal physiology and NNT deficiency manifests with adrenal insufficiency in patients (Roucher-Boulez et al., 2016; Meimaridou et al., 2018). This indicates that while NNT serves to supplement the NADPH pool across tissue types (Lopert and Patel, 2014; Fisher-Wellman et al., 2015; Ronchi et al., 2016; Meimaridou et al., 2018), its functional contribution to redox homoeostasis may vary, especially with regards to malignancy (Gameiro et al., 2013; Ho et al., 2017; Chortis et al., 2018; Li et al., 2018).

It has been shown in disparate cancer cell lines that disruption of redox homeostasis following the loss of NNT expression induces metabolic rewiring, marked by changes in fuel utility (Gameiro et al., 2013; Ho et al., 2017). In an endothelial cell line derived from the ascitic fluid of a patient with liver adenocarcinoma, NNT knockdown was associated with reduced flux of both glucose and glutamine carbon through the TCA cycle coupled with a shift toward reductive glutamine metabolism (Ho et al., 2017). This is in contrast to melanoma cells, which exhibit increased glucose flux through the TCA cycle at the expense of reductive glutamine metabolism (Gameiro et al., 2013). This finding aligns with a study by Mullen et al. (2014), which demonstrated a connection between NNT-derived NADPH and reductive glutamine metabolism. Though we did not assess carbon flux through the TCA cycle, our data indicate a reduced capacity to oxidize either glucose or glutamine carbon in NSCLC cells following NNT knockdown (Figs. 3 and S2). The divergence in metabolic consequences following disruption of NNT expression in various cancers further suggests a context dependency to its function.

Collectively, our results indicate that NNT influences the activity of several Fe-S proteins critical to mitochondrial metabolism. This is most apparent in H1299, H2009, and PC9 cells, while the effect in A549 cells is less severe. This agrees with our initial evaluation of mitochondrial function, where NNT knockdown had a less pronounced influence on maximal respiratory capacity in A549 cells compared with the more sensitive PC9 cells (Fig. 3 A). Similar to H441 cells, A549 cells may rely upon other sources of mitochondrial NADPH more than NNT. Through our liquid chromatography/mass spectrometry–based assessment of the impact of NNT loss on NSCLC metabolism, we revealed a dysregulation of mitochondrial metabolism in H1299, H2009, and PC9 cells, marked by a severe depletion of most TCA cycle intermediates (Fig. 5 D). This indicates that the diminished function of mitochondrial Fe-S proteins associated with NNT loss does elicit a severe metabolic defect. While the impact of NNT loss on Fe-S protein function was strikingly similar to that of disrupting the Fe-S cluster biosynthetic machinery, there were distinct effects on the TCA cycle that distinguished NNT loss from ISCU knockdown (Fig. 5). This deviation is potentially attributable to the influence of ISCU but not NNT on protein lipoylation. Unlike the clusters associated with ACO2 or the respiratory chain, the 4Fe-4S cluster that mediates LIAS catalysis is consumed in the process to contribute sulfur for synthesis of lipoic acid (Parry and Trainor, 1978; Miller et al., 2000; Crooks et al., 2018). This activity permits lipoylation of components of PDH and α-ketoglutarate dehydrogenase, which are required for TCA cycling. The lack of a discernable effect on the lipoylation status of PDH and DLST following NNT knockdown indicates that NNT does not influence these proteins in the same manner as ISCU (Fig. 5 E). Still, the metabolic effects of NNT loss were very reminiscent of those elicited by the acute loss of Fe-S clusters, which included an accumulation of fatty acids (Crooks et al., 2018). In that context, an ISCU deficiency promoted de novo fatty acid synthesis from citrate carbon that could not be efficiently oxidized in the TCA cycle due to the corresponding loss of Fe-S protein function. It is unlikely that NNT loss promotes fatty acid synthesis in NSCLC cells due to the significant demand for NADPH in the generation of fatty acids and the observed decrease in NADPH availability associated with NNT knockdown (Fig. 2 D). Indeed, we observe indications of this in NNT-deficient cells starved of serum fatty acids (Fig. 6 H). This accumulation of fatty acids is likely a result of increased uptake (Fig. 6 D), which occurs in NSCLC cells incapable of de novo synthesis (Migita et al., 2008). Regardless, fatty acid accumulation is a shared response to the disruption of either NNT or ISCU expression. The cytotoxic effects of both fatty acid supplementation and starvation in NNT-deficient cells (Fig. 6, E–G) is highly intriguing and suggests an important role for NNT in fine-tuning fatty acid metabolism that requires further study. This also indicates a varied and complex role for NNT activity in regulating NSCLC metabolism that extends beyond our findings regarding Fe-S protein function.

The differential effects of NNT loss on mitochondrial Fe-S proteins with respect to the catalytic function of the resident cluster(s) suggested that NNT may influence cluster integrity rather than availability. Fe-S clusters are exquisitely sensitive to oxidation, including by molecular oxygen (Crack et al., 2014; Alvarez et al., 2017). Cluster oxidation is associated with dislocation of an iron atom that renders the cluster inactive (Djaman et al., 2004). The highly oxygenated pulmonary environment likely dictates the substantial mitochondrial oxidation exhibited by human lung tumors (Hensley et al., 2016), as oxidative phosphorylation exploits the detoxification of molecular oxygen for an energetic benefit. Interestingly, NFS1 is positively selected for in NSCLC and is shown to support sustained Fe-S cluster biosynthesis to protect against the excessive oxygen challenge associated with residency in the lung (Alvarez et al., 2017). These clusters are also subject to oxidation by the reactive products of the same metabolism that protects against the accumulation of molecular oxygen (Flint et al., 1993; Djaman et al., 2004). Though we do not observe a substantial induction of ROS associated with NNT knockdown (Figs. 2 E and S1 B), we are able to rescue the effects of NNT loss on Fe-S protein function with MitoCatalase and other antioxidants that affect mitochondrial antioxidant capacity (Figs. 7 and S5). Moreover, the differential effect of NNT and ISCU loss on LIAS function may further reflect the importance of NNT to cluster integrity rather than synthesis. Given that the cluster associated with LIAS must be consistently turned over due to its consumption during catalysis, it is likely less prone to oxidation.

Considering our similar ability to rescue Fe-S protein function with pos5p (Fig. 5), our collective data indicate a potential role for NNT-derived NADPH in the mitigation of Fe-S cluster oxidation. This is intuitive considering that NNT is localized to the same membrane as the respiratory chain, which is a significant source of mitochondrial ROS. Thus, NNT may provide a regionalized source of reducing power to protect the integrity of the ETC. With regards to the potential impact of NNT activity on the soluble ACO2, there is substantial evidence that ACO2 physically associates with TCA cycle enzymes to form dynamic assemblies to enhance substrate channeling (Porpaczy et al., 1987; Morgunov and Srere, 1998; Fernie et al., 2018). Moreover, several of the NADH yielding dehydrogenases associate with the IMM, likely enhancing oxidation by complex I (D’Souza and Srere, 1983; Sumegi and Srere, 1984a, b). Together, this suggests a likely spatial association between the TCA cycle machinery and the IMM permitting regulation by NNT. Indeed, there is existing evidence that NNT contributes to a redox cycle with PDH (Fisher-Wellman et al., 2015). As described, NNT consumes the NADH yielded by PDH to generate the NADPH required to support the detoxification of H_2_O_2_ that is also produced as a byproduct of PDH catalysis (Fisher-Wellman et al., 2013). This NNT-dependent redox circuit was linked to respiratory capacity and energy expenditure in mice (Fisher-Wellman et al., 2015).

Altogether, our study demonstrates that NNT is of significance to lung tumor biology, in part through the regulation of Fe-S proteins that facilitate mitochondrial metabolism. In contrast to previous studies evaluating NNT function, we describe a nuanced influence on mitochondrial redox homeostasis in NSCLC, where NNT activity likely mitigates regionalized oxidative stress stemming from the substantial oxidative metabolism exhibited by lung tumors. Our findings further indicate a necessity for mitochondrial metabolism in lung tumorigenesis, underscoring the therapeutic potential of augmenting mitochondrial function (Weinberg and Chandel, 2015).

## Materials and methods

### Mice

*LSL-Kras^G12D^* mice (Jackson et al., 2001) on a C57BL6/J background were crossed with 129/Svj (*Nnt^+/+^*) mice to breed in a functional *Nnt* allele. All experimental mice are derived from this F1 generation and are from the F2–F4 generations. All *LSL-Kras^G12D^*; *Nnt^+/+^* and *LSL-Kras^G12D^*; *Nnt^Δex7-11/Δex7-11^* experimental mice were littermates. *LSL-Kras^G12D^* and *Trp53^flox^* mice (Jackson et al., 2005) on a C57BL6/129 mixed background were crossed with C57BL/6J mice to obtain the *Nnt^Δex7-11/Δex7-11^* allele. These F1 mice were interbred to generate cohorts of *LSL-Kras^G12D^; Trp53^flox/flox^; Nnt^Δex7-11/Δex7-11^*, *LSL-Kras^G12D^; Trp53^flox/flox^; Nnt^Δex7-11/+^*, and *LSL-Kras^G12D^; Trp53^flox/flox^; Nnt^+/+^* mice for lung tumor studies. All mice were genotyped to confirm Nnt alleles with previously defined primers (Navarro et al., 2012). The Nnt Common primer (5′-GTA​GGG​CCA​ACT​GTT​TCT​GCA​TGA-3′) was used in the amplification of both the wild-type (*Nnt^+^*) and mutant (*Nnt^Δex7-11^*) alleles, whereas specific wild-type (5′-GGG​CAT​AGG​AAG​CAA​ATA​CCA​AGT​TG-3′) and mutant (5′-GTG​GAA​TTC​CGC​TGA​GAG​AAC​TCT​T-3′) primers were used to amplify their respective alleles. To induce lung tumor formation, mice under isofluorane anesthesia were infected intranasally with 10^7^ PFU of adenoviral-Cre (University of Iowa, Iowa City, IA) as previously described (Jackson et al., 2001). All mice studies were approved by and conducted in accordance to the ethical standards established by the University of South Florida Institutional Animal Care and Use Committee (protocol no. R IS00003893).

### Tumor analysis

For analyses of Kras^G12D^; Nnt^Δex7-11/Δex7-11^ and Kras^G12D^; Nnt^+/+^ tumors, mice were euthanized 3 mo following tumor induction with adenoviral-Cre. Lungs were collected and fixed in 10% formalin overnight and then embedded in paraffin for subsequent sectioning. Sections were deparafinized in xylene and then rehydrated in a graded series of ethanol solutions. Slides were then sequentially stained with H&E, dehydrated in ethanol and xylene, coverslipped, and then dried overnight. Slides were scanned with the Aperio imager (Leica Biosystems), and each lung specimen was analyzed with ImageScope (Aperio). For analyses of Kras^G12D^; p53^Δ/Δ^; Nnt^Δex7-11/Δex7-11^, Kras^G12D^; p53^Δ/Δ^; Nnt^Δex7-11/+^, and Kras^G12D^; p53^Δ/Δ^; Nnt^+/+^ tumors, mice were enrolled in a survival study and allowed to reach an experimental endpoint in agreement with ethical standards. Lung specimens were collected, H&E slides were generated, and histopathology analysis was performed to grade lesions on a (1–5) scale based on criteria previously established for this model (Jackson et al., 2005; DuPage et al., 2009).

### Lentiviral generation and infection

Lenti-X 293 T cells (Clontech) at 90% confluency were cotransfected with the plasmid of interest and the packaging plasmids pCMV-dR8.2 dvpr (Addgene, 8455) and pCMV-VSV-G (Addgene; 8454) in the presence of JetPRIME (Polyplus). Cells were infected with lentivirus and 8 µg/ml polybrene for 6 h at an optimized dilution established with the Lenti-X GoStix Plus system (Takara).

### Cell lines and culture

Human lung adenocarcinoma cell lines (DeNicola et al., 2015) were maintained in RPMI 1640 medium (Hyclone or Gibco) supplemented with 10% FBS and in the absence of antibiotics at 37°C in a humidified incubator containing 95% air and 5% CO_2_. Cell lines were routinely tested for mycoplasma contamination with the MycoAlert Assay (Lonza). ISCU (Addgene, 102972), NFS1 (Addgene, 102963, 102964), and NNT (Open Biosystems, TRCN0000028507, TRCN0000028512) knockdown was achieved using validated short hairpin sequences targeting these transcripts in a pLKO.1 vector, a nontargeting scrambled sequence (Millipore Sigma, SHC002) was used as a negative control. Cells were infected with shRNA or scramble lentivirus and then selected with 1 µg/ml puromycin for 3 d before each experiment. All experiments were conducted 4 d after lentiviral infection except for analyses of proliferation or viability. The Pos5p nucleotide sequence was purchased as a gBlock from Integrated DNA Technologies. Pos5p was modified to include flanking 3X HA-tags and cloned into the pLenti-CMV-Blast vector (Addgene, 17445). Cells were infected with either pos5p or GFP control lentivirus and then selected with 10 µg/ml blasticidin for 5 d. Similar to the strategy used to generate a mitochondrially targeted catalase-expressing mouse (Schriner et al., 2005), the initiating methionine codon of human catalase cDNA was replaced with the sequence for the first 25 amino acids of the ornithine transcarbamylase leader sequence to target exogenous catalase protein to the mitochondria. The MitoCatalase sequence was cloned into the pPHAGE C-TAP vector (Huttlin et al., 2015). Cells were infected with either MitoCatalase or luciferase (Luc) control lentivirus and then selected with 1 µg/ml puromycin for 5 d.

### Analyses of cell proliferation and viability

For proliferation assays, cells were seeded in triplicate on four 96-well plates at a density of 2,500–5,000 cells/well in 100 µl. The next day, cells were infected with scramble or NNT-targeting shRNA lentivirus in a final volume of 50 µl, then overlaid with 100 µl of medium and allowed to proliferate. On day 1 after infection, one of the plates was collected and the cells fixed with 4% paraformaldehyde. This was repeated on days 3, 5, and 7 after infection. Fixed cells were then stained with crystal violet, washed, and dried overnight. Crystal violet was solubilized with 100 µl of 10% acetic acid, and the OD_600_ was measured with a spectrophotometer (Promega). For viability assays, cells were plated on 96-well plates at a density of 10,000 cells/well in 100 µl. On the next day, cells were incubated with 100 µl of media containing auranofin (Sigma-Aldrich), tbHP (Sigma-Aldrich), CHP (Invitrogen), menadione (Thermo Fisher Scientific), BSO (Cayman Chemical), 6-aminonicotinamide (Alfa Aesar), oleate (Thermo Fisher Scientific), or palmitate (Sigma-Aldrich) at the indicated concentrations for 24 h. Cells were then fixed with 4% paraformaldehyde, stained with crystal violet, washed, and dried overnight. Crystal violet was solubilized with 100 µl of 10% acetic acid and the OD_600_ determined. For experiments evaluating the effect of NNT knockdown, cells were seeded on day 2 after infection. Relative viability was determined following normalization to scramble or DMSO-treated cells.

Alternatively, cell viability was determined 4 or 7 d after lentiviral infection with the MultiTOX-Glo Multiplex Cytotoxicity Assay (Promega). In brief, 30,000 puromycin-selected cells were transferred in 50 µl to triplicate wells of a white 96-well luminescence microplate. 25 µl of glycylphenylalanyl-aminofluorocoumarin reagent was added to each well, the plate was covered with foil to protect from light, and the cells were incubated at 37°C for 1 h. The fluorescence signal was then determined (excitation: 400 nm; emission: 500–550 nm) with a fluorescence-compatible plate reader. 25 µl of the AAF-Glo reagent was then added to each well, and the plate incubated in the dark at room temperature for 15 min. The luminescence signal was then determined with a luminescence-compatible plate reader. The fluorescent signal is representative of the live cell population within the well and the luminescent signal representative of the population of dead cells; the ratio of these signals was determined for each well and the relative viability determined following normalization to scramble infected cells.

### Immunoblotting

Cell lysates were generated in ice-cold RIPA buffer (20 mM Tris-HCl, pH 7.5 [VWR], 150 mM NaCl [Thermo Fisher Scientific], 1 mM EGTA [VWR], 1 mM EDTA [Sigma-Aldrich], 1% sodium deoxycholate [Sigma-Aldrich], 1% NP-40 [Sigma-Aldrich]) supplemented with protease inhibitors (Roche). Protein concentrations were determined by DC Protein Assay (Bio-Rad) before mixing with a 6× reducing sample buffer containing β-mercaptoethanol (VWR). Proteins were separated by SDS-PAGE using NuPAGE 4–12% Bis-Tris precast gels (Invitrogen), then transferred to 0.45 µM nitrocellulose membranes (GE Healthcare). For analyses of PRDX1 and PRDX3 oxidation, a previously published protocol was followed (Cox et al., 2009). These redox Western samples were mixed with a 4× nonreducing buffer before separation by SDS-PAGE. For analyses of TXN1 oxidation, a previously established, urea-PAGE protocol was followed (Du et al., 2013; Harris et al., 2019). Two control lysates were fully reduced with dithiothreitol (VWR) and then treated with iodoacetic acid (Sigma-Aldrich) or iodoacetamide (Sigma-Aldrich) to represent fully oxidized or reduced TXN protein. Following urea-PAGE, these samples were also transferred to nitrocellulose membranes.

Membranes were blocked with 5% nonfat milk in Tris-buffered saline with 0.1% Tween 20 and then incubated with the following primary antibodies: ACO2 (GeneTex, GTX109736), Actin (Thermo Fisher Scientific, AC-15), Catalase (Cell Signaling Technologies, D4P7B), DLST (Cell Signaling Technologies, D22B1), HA-Tag (Cell Signaling Technologies, C29F4), HSP90 (Cell Signaling Technologies, 4874S), ISCU (Santa Cruz Biotechnology, sc-373694), lipoic acid (Millipore Sigma, 437695), NFS1 (Santa Cruz Biotechnology, sc-365308), NNT for cell lysates (Abcam, ab110352), NNT for mouse tissue lysates (GeneTex, GTX103015), NQO1 (Sigma-Aldrich, HPA007308), NRF2 (Cell Signaling Technologies, D1Z9C), PDH-E2 (Abcam, ab126224), PRDX1 (Cell Signaling Technologies, D5G12), PRDX3 (Abcam, ab73349), TRXR1 (Cell Signaling Technologies, D1T3D), TXN1 (Cell Signaling Technologies, C63C6), TXN2 (Sigma-Aldrich, HPA000994), and α-tubulin (Santa Crux Biotechnology, sc-8035). HRP-conjugated secondary antibodies and enhanced chemiluminescence were used for all immunoblotting.

### Flow cytometry analyses of ROS

For all analyses of ROS, 10^5^ cells were seeded overnight in triplicate wells of a 6-well plate. Mitochondrial H_2_O_2_ levels were determined using the fluorescent dye MitoPy1 (Tocris) according to an established protocol (Dickinson et al., 2013). Briefly, cells were incubated in fresh media for 4 h, washed with PBS (Hyclone or Sigma-Aldrich), and incubated in 1 ml of 10 µM MitoPY1 for 30 min. Depending on the experiment, cells were either collected immediately for analysis or challenged with H_2_O_2_ for an additional 30 min before collection. Mitochondrial •O_2_^−^ levels were determined with the fluorescent dye MitoSox Red (Invitrogen) according to an established protocol (Kauffman et al., 2016). Briefly, cells were incubated in fresh media for 4 h, washed with PBS, and incubated in 1 ml of 5 µM MitoSOX Red in HBSS (Gibco) for 20 min. Cells were then collected immediately for analysis. Cytosolic ROS levels were determined with the fluorescent dye CellRox Green (Invitrogen) according to the manufacturer’s protocol. Briefly, cells were incubated in 1 ml of fresh media for 4 h, at which point 2 µl of 2.5 mM CellROX Green was added to each well for a final concentration of 5 µM. Cells were incubated with CellROX green for 30 min and then collected for analysis. The fluorescence of dye-loaded cells was determined by flow cytometry with a BD Biosciences 2 Laser 4 Color FacsCalibur Flow Cytometer (Marshall Scientific). The FL1 channel was used for analyses of MitoPY1 and CellROX Green fluorescence, whereas the FL3 channel was used for analyses of MitoSOX Red fluorescence. The mean fluorescence intensity of 10,000 discrete events was calculated for each sample.

### NADPH:NADP^+^ assay

25,000 cells were seeded in 500 µl of medium overnight in triplicate in 12-well plates. Cells were then incubated in fresh media for 4 h, collected, and extracted to determine the NADPH/NADP^+^ ratio according to the NADP/NADPH-Glo Assay Kit (Promega) protocol.

### Seahorse analyses of metabolic function

All measures of oxygen consumption and extracellular acidification were determined with a Seahorse XFe96 Analyzer (Agilent). General mitochondrial function was assessed according to the Seahorse XF Cell Mito Stress Kit protocol (Agilent). Metabolic phenotyping was assessed according to the Seahorse XF Cell Energy Phenotype Kit protocol (Agilent). Briefly, basal OCR and ECAR rates were determined in the presence of 10 mM glucose (Sigma-Aldrich), 2 mM glutamine (Thermo Fisher Scientific), and 1 mM pyruvate (Sigma-Aldrich). Cells were then subjected to the simultaneous application of 1 mM oligomycin (Sigma-Aldrich) and indicated concentrations of carbonyl cyanide 4-(trifluoromethoxy)phenylhydrazone (FCCP; Sigma-Aldrich) to determine OCR and ECAR under stressed conditions. Glycolytic capacity was determined according to the Seahorse XF Glycolysis Stress Test Kit protocol (Agilent). Assessments of individual respiratory chain activities were performed according to a previously established protocol (Salabei et al., 2014). Briefly, 40,000 cells were plated in quadruplicate on an XFe96 microplate and allowed to seed overnight. Immediately before assay, cells were overlaid with 175 µl of mitochondrial assay solution (220 mM mannitol [Sigma-Aldrich], 70 mM sucrose [Sigma-Aldrich], 10 mM KH_2_PO_4_ [VWR], 5 mM MgCl_2_ [VWR], 2 mM Hepes [Thermo Fisher Scientific], and 1 mM EGTA [VWR]) supplemented with the Seahorse Plasma Membrane Permeabilizer (Agilent), 4 mM ADP (Sigma-Aldrich), and either 10 mM sodium pyruvate (Sigma-Aldrich) and 1 mM malate (Sigma-Aldrich) or 10 mM glutamate (Sigma-Aldrich) and 1 mM malate. Cells were then sequentially subjected to 2 µM rotenone (Sigma-Aldrich), 10 mM succinate (Sigma-Aldrich), 2 µM antimycin A (Sigma-Aldrich), and 10 mM ascorbate (Sigma-Aldrich) with 100 µM N,N,N′,N′-tetramethyl-ρ-phenylene diamine (Sigma-Aldrich). Last, palmitate oxidation was determined using the XF Fatty Acid Oxidation Assay Kit (Agilent) protocol.

### Aconitase assay

Aconitase activity was determined based on a modified version of a protocol previously described (Francisco et al., 2018). Cells were collected and resuspended in 250 µl of 50 mM Tris-HCl and 150 mM NaCl, pH 7.4. The cell suspension was homogenized with a dounce homogenizer and the homogenate spun down for 10 min at 10,000 *g* at 4°C. The pellet was then washed twice and resuspended in 100 µl of 1% Triton X-100 (Sigma-Aldrich) in 50 mM Tris-HCl, pH 7.4, to lyse the mitochondrial membrane. This fraction was then spun down for 15 min at 17,000 *g* at 4°C. The protein concentration was then determined by DC Protein Assay (Bio-Rad), and 175 µl of 100–500 μg/ml protein solution was generated with assay buffer (50 mM Tris-HCl, pH 7.4). 50 µl of this solution was transferred to triplicate wells of a black-walled 96-well fluorescence microplate already containing 55 µl of assay buffer. Next, 50 µl of a 4 mM NADP^+^ (Sigma-Aldrich), 20 U/ml IDH1 (Sigma-Aldrich) solution was added to each well. Finally, 50 µl of 10 mM sodium citrate (Sigma-Aldrich) was added to each well to initiate the assay. The plate was transferred to a fluorescence-compatible plate reader (Promega) to measure NADPH autofluorescence every minute over a period of an hour. This change in fluorescence over time is indicative of aconitase activity, where ACO2 present in the mitochondrial protein fraction converts the supplied citrate to isocitrate, which the supplied IDH1 then metabolizes in a reaction that generates NADPH. For experiments assessing the effect of exogenous antioxidants on ACO2 activity, lentiviral-infected cells were subjected to 24-h treatment with 2 mM NAC or 25 nM MitoTEMPO before assay. Alternatively, for experiments assessing the effect of recombinant catalase (Sigma-Aldrich), lentiviral-infected cells were incubated overnight with 1,000 U of catalase before the assay (Huang et al., 2012; Bey et al., 2013). For the analysis of tumor tissue, tumors were homogenized in 500 µl of mitochondrial isolation buffer (200 mM mannitol, 10 mM sucrose, 1 mM EGTA, and 10 mM Hepes, pH 7.4). Homogenates were spun down for 10 min at 800 *g* at 4°C. The supernatant was subjected to an additional spin of 10,000 *g* at 4°C. The pellet was then processed for analysis as described.

### LC-HRMS metabolomics

NSCLC cells seeded in triplicate wells of a 6-well plate were rapidly washed in ice-cold PBS and extracted in 0.5 ml of 80% methanol overnight at –80°C. Extracts were then cleared by centrifugation (17,000 *g* for 30 min at 4°C), and the supernatant analyzed by LC-HRMS. We performed this LC-HRMS analysis under the conditions for nontargeted metabolomics that we have established previously (Kang et al., 2019). Briefly, we used a Vanquish UPLC system coupled to a Q Exactive HF mass spectrometer equipped with heated electrospray ionization (Thermo Fisher Scientific). A SeQuant ZIC-pHILIC LC column, 5 µm, 150 × 4.6 mm (Millipore Sigma) with a SeQuant ZIC-pHILIC guard column, 20 × 4.6 mm (Millipore Sigma) was used for chromatographic separation. The mobile phase A consisted of 10 mM ammonium carbonate and 0.05% ammonium hydroxide in water, while mobile phase B was 100% acetonitrile. The MS1 scan was operated in both positive and negative modes for data acquisition, and data were analyzed with El Maven v0.3.1 (Clasquin et al., 2012). Metabolite identification was based on a comparison of both the mass-to-charge (m/z) value and retention time of sample peaks to an internal library (Mass Spectrometry Metabolite Libarary of Standards, Sigma-Aldrich).

### Analysis of fatty acid uptake

NSCLC cells were seeded overnight in triplicate wells of a black-walled 96-well fluorescence microplate. Cells were then washed twice and overlaid with RPMI supplemented with 10% delipidated FBS for 4 h. Briefly, delipidated serum was generated by adding 2 g of fumed silica (Sigma-Aldrich) to 100 ml of FBS and mixed overnight at room temperature. Serum was then spun down at 2,000 *g* for 15 min and the supernatant collected and filtered. Following incubation with delipidated media, cells were washed with PBS and incubated with 10 µM BODIPY-FL-C16 (Invitrogen) for 30 min. Cells were then washed and overlaid with PBS, and their fluorescence determined with a fluorescence-compatible plate reader.

### Statistical analysis

Data were analyzed for statistical significance with GraphPad Prism 8 software. Values of P < 0.05 were considered significant (n.s., not significant; *, P < 0.05; **, P < 0.01; ***, P < 0.001; ****, P < 0.0001). Differences between survival curves were determined by the log-rank test. Comparisons of two groups were performed with a two-sided unpaired Student’s *t* test. A one-way ANOVA with a post hoc Brown–Forsythe test was performed for comparisons of three or more groups, as similar variances between groups were observed. Data are reported as mean ± SD of at least three technical replicates and representative of at least three experimental replicates unless noted otherwise. In assessing the effects of NNT knockdown in GFP/pos5p and Luc/MitoCatalase cells, a two-way ANOVA with a Sidak’s multiple comparisons test was performed.

### Online supplemental material

Fig. S1 shows additional analyses of mitochondrial oxidative stress and the response to exogenous oxidant treatment in NSCLC cells subject to NNT knockdown. Fig. S2 shows analyses of cytosolic oxidative stress in NSCLC cells subject to NNT knockdown. Fig. S3 shows additional measures of metabolic function in NSCLC cells in addition to immunoblot analyses of ACO2 expression in NNT-deficient NSCLC cells and KP tumors with differential Nnt expression. Fig. S4 shows analyses of basal mitochondrial function in pos5p-expressing NSCLC cells. Fig. S5 demonstrates the functionality of MitoCatalase in MitoCatalase-expressing NSCLC cells and the impact of alternative antioxidants on ACO2 activity in NNT-deficient cells.
